# Key enzymes catalyzing glycerol to 1,3-propanediol

**DOI:** 10.1186/s13068-016-0473-6

**Published:** 2016-03-10

**Authors:** Wei Jiang, Shizhen Wang, Yuanpeng Wang, Baishan Fang

**Affiliations:** Department of Chemical and Biochemical Engineering, College of Chemistry and Chemical Engineering, Xiamen University, Xiamen, 361005 China; The Key Lab for Synthetic Biotechnology of Xiamen City, Xiamen University, Xiamen, 361005 China; The Key Laboratory for Chemical Biology of Fujian Province, Xiamen University, Xiamen, 361005 Fujian China

**Keywords:** Bioconversion, Biodiesel, Glycerol, Biocatalyst, GDHt, GDH, PDOR, Industrial enzyme, Multienzyme coupling, Renewable resources

## Abstract

Biodiesel can replace petroleum diesel as it is produced from animal fats and vegetable oils, and it produces about 10 % (w/w) glycerol, which is a promising new industrial microbial carbon, as a major by-product. One of the most potential applications of glycerol is its biotransformation to high value chemicals such as 1,3-propanediol (1,3-PD), dihydroxyacetone (DHA), succinic acid, etc., through microbial fermentation. Glycerol dehydratase, 1,3-propanediol dehydrogenase (1,3-propanediol-oxydoreductase), and glycerol dehydrogenase, which were encoded, respectively, by *dhaB*, *dhaT*, and *dhaD* and with DHA kinase are encompassed by the dha regulon, are the three key enzymes in glycerol bioconversion into 1,3-PD and DHA, and these are discussed in this review article. The summary of the main research direction of these three key enzyme and methods of glycerol bioconversion into 1,3-PD and DHA indicates their potential application in future enzymatic research and industrial production, especially in biodiesel industry.

## Background

The demand for biofuels is increasing worldwide; as raw material, it is one of the most promising alternative sources of energy. Biodiesel can replace petroleum diesel as it is derived from vegetable oils and animal fats, and it produces about 10 % (w/w) glycerol as a major byproduct [1–6]. However, as glycerol cannot be disposed of in the environment safely, surplus glycerol generated could become an environmental threat. In the EU, some biodiesel companies face many serious problems with regard to the removal of excess glycerol, and the disposal of glycerol is quite expensive. Collapse of the price of glycerol is the main problem faced by these companies [7–11]. One of the promising applications of glycerol is its bioconversion to 1,3-propanediol (1,3-PD) [12–15], which is a valuable chemical and one of the six new petrochemical products, as it can be used as a monomer for polycondensation to manufacture plastics with special properties, i.e., polyesters, polyethers, polyurethanes, polytrimethylene terephthalate [16–24], as a monomer for cyclic compounds [25, 26], and as a polyglycol-type lubricant. Besides, it may also serve as a solvent [27, 28]. In the market for these new products, exploiting low-cost 1,3-PD will be the key to its competitiveness.

**Fig. 1:**
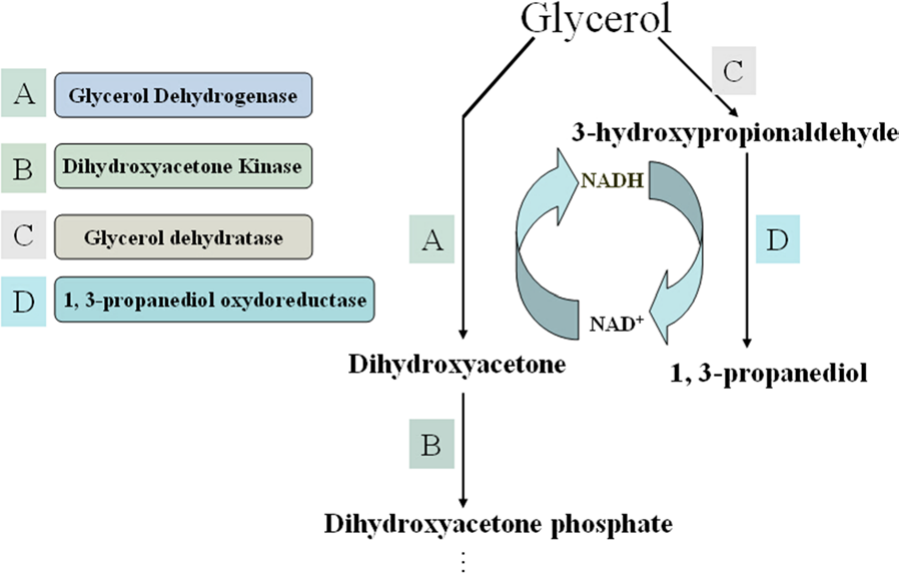
Main Pathway for bioconversion glycerol to 1, 3-propanediol

More than 10^5^ tons of 1,3-PD are produced every year. Most of them are obtained through chemical synthesis, but this process is expensive and environmentally hazardous as it requires high temperature, high pressure, expensive catalysts, and produces toxic intermediates; hence, 1,3-PD still has a low market volume on industrial scale [29–36]. In contrast, the biotechnological synthesis of 1,3-PD appears to be a promising alternative to the chemical synthesis as it offers environmental benefits and allows for the use of renewable feedstock [37, 38]. 1,3-PD was originally obtained from glycerol fermentation in 1881 [39]. Glycerol is metabolized both oxidatively and reductively in *Citrobacter*, *Klebsiella*, *Enterobacter*, *Clostridium*, etc. [40, 41]. The pathway of conversion from glycerol to 1,3-PD is shown in Fig. 1. More studies on the three strains, *K. pneumoniae*, *C. butyricum*, and *C. freundii*, have been performed than on other species, as these species have high rates of conversion to 1,3-PD and great production intensities.

1,3-PD is most likely to become the most widely used bulk chemical produced from a renewable resource with biological technology [42]. In recent years, the interest has been focused on the production of 1,3-PD using enzymatic conversion. The reductive pathway is regulated and controlled by coenzyme B_12_-dependent glycerol dehydratase (GDHt) (EC, 4.2.1.30) and related diol dehydratases (DDHs) (EC 4.2.1.28) [25, 43–45], converting glycerol to 3-hydroxypropionaldehyde (HPA) [46–49], and by the coenzyme nicotinamide adenine dinucleotide (NADH)+H^+^-dependent enzyme, 1,3-propanediol-oxydoreductase (PDOR) (EC 1.1.1.202), reducing 3-HPA to 1,3-PD and regenerating NAD^+^ [30, 50–54] (Fig. 1). In the oxidative pathway, the NAD^+^-dependent enzyme glycerol dehydrogenase (GDH) (EC 1.1.1.6) catalyzes the conversion of glycerol to dihydroxyacetone (DHA), then the glycolytic enzyme DHA kinase (EC 2.7.1.29, dhaK) phosphorylates the DHA to the latter product [51, 55–57], which is then funneled to glycolysis (Fig. 1). GDH, GDHt, and PDOR, which were encoded, respectively, by *dhaB*, *dhaT*, and *dhaD* and with dhaK are encompassed by the dha regulon [40, 44, 45], are the three key enzymes in the bioconversion of glycerol in 1,3-PD and DHA. Furthermore, these are discussed in this review article.

## Glycerol dehydratase

GDHt, which catalyzes the penultimate step in the fermentation pathway to produce 1,3-PD [29, 44, 46], is a key and rate-limiting enzyme for the conversion of glycerol to 3-HPA. 3-HPA is further reduced to 1,3-PD by the NADH-linked PDOR. Furthermore, the genes of the GDHt are located in the DHA regulon [58, 59]. Mainly, the GDHt, which is mostly found in *C. freundii*, *K. pneumoniae*, *C. pasteurianum*, etc. [51, 58, 60, 61], consists of three types of subunits, i.e., α, β, and γ, and exists in the form of α_2_β_2_γ_2_ heterohexamer.

**Fig. 2:**
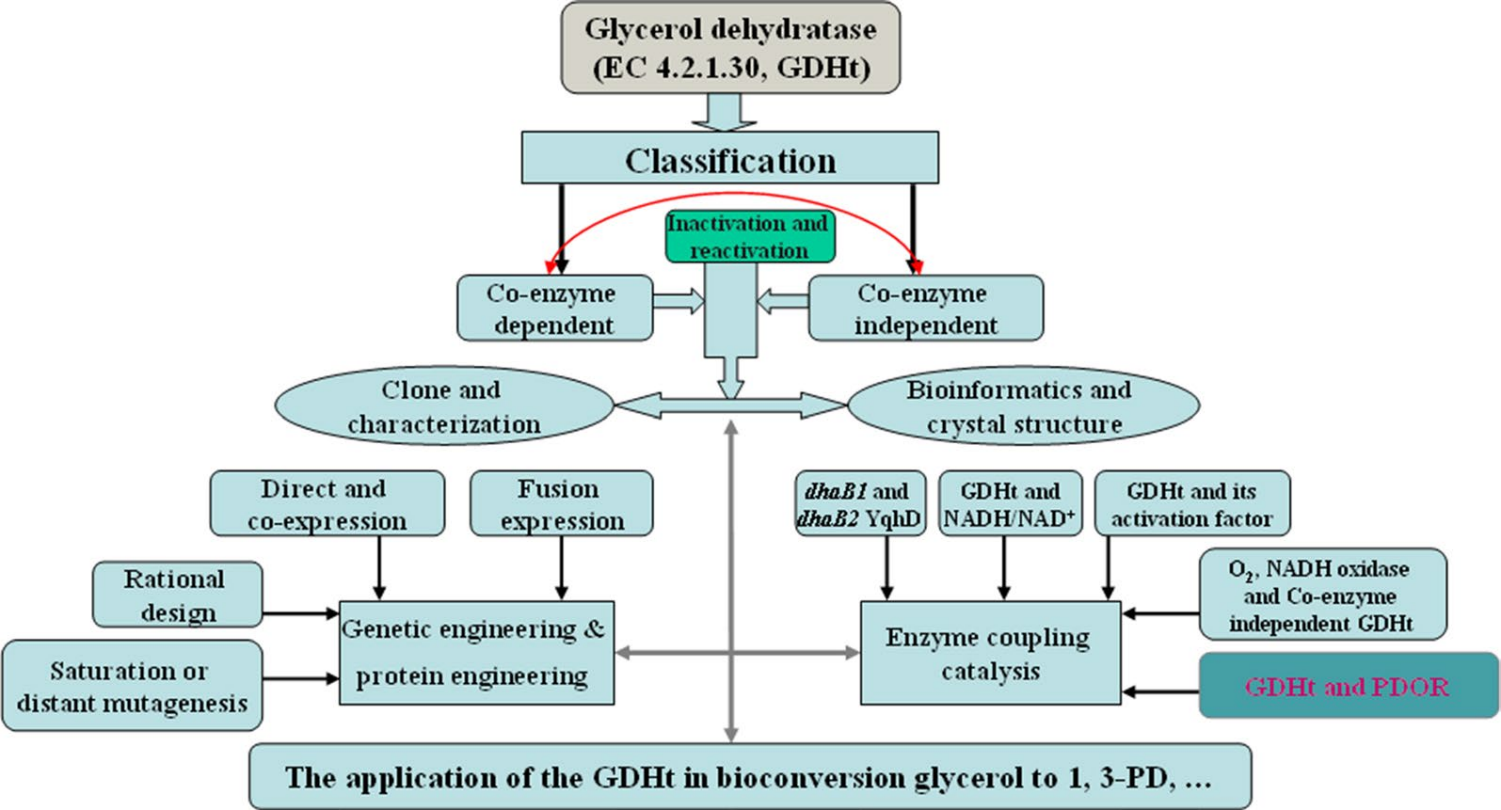
Summary of main research direction about GDHt

The 1,3-PD operon of *C. butyricum* comprises three genes, a different type of GDHt (*dhaB1*), its activator protein (*dhaB2*), and *dhaT* [33]. In this bacterium, GDHt is extremely oxygen sensitive, strongly associated with the cell membrane, and independent of vitamin B_12_ [33–35, 62–64]. The GDHt limits the activity of the propanediol dehydrogenase [65]. Moreover, the GDHt is a key functional molecule in the catabolism of glycerol by *C. butyricum*. Hence, this rate-limiting step avoids intracellular accumulation of 3-HPA, a very toxic compound. A summary of main research direction and research content of GDHt is shown in Fig. 2.

### Classification of GDHt

GDHt and DDH from 1,3-PD-producing bacteria can be categorized into three categories: the first one, which is distributed on the cell membrane, does not rely on the coenzyme B_12_, is sensitive to oxygen, and is representative of ethylene glycol *C. glycolycum* of DDH; the second relies on the coenzyme B_12_, shows resistance to oxygen, undergoes suicidal inactivation by substrate glycerol and is represented by GDHt of *K. pneumoniae*, *Citrobacter*, and *C. pasteurianum* of the GDHt (Fig. 2); the third class does not rely on the coenzyme B_12_, is sensitive to oxygen, undergoes suicidal inactivation by the substrate glycerol, activated again with the help of SAM, and is representative of *C. butyricum* in the GDHt (Fig. 2).

The differences between GDHt and DDH were shown by comparing the three-dimensional structures of GDHt to that of DDH [66–68]. It was demonstrated that the substrate bound to GDHt was assigned the (*R*)-isomer, which is the difference between the two enzymes for suicidal inactivation of sensitivity by glycerol [66]. A new type of GDHt shows no homology with the well-known B_12_-dependent GDHt, but shows significant similarity with the pyruvate formate lyases (PFLs). PFLs activate enzymes and their homologous compounds, which are encoded by the *dhaB1* from *C. butyricum*, were cloned and analyzed [33], and they were proved to be belonging to a new family of coenzyme B_12_-independent GDHt. The enzyme is different from the coenzyme B_12_-dependent dehydratases in that it (i) is extremely oxygen sensitive, (ii) is strongly associated with the cell membrane, and (iii) does not use cobalamin coenzyme as a cofactor [69]. The genes encoding the 1,3-PD operon of *C. butyricum* VPI1718 comprises three genes: *dhaB1*, *dhaB2,* and *dhaT*, which encode a new type of GDHt, its activator protein and PDOR, respectively. In our group, through the expressing recombinant expression vector pET-22 *dha*B_1_B_2_ in *Escherichia coli* BL21 (DE3), the activity of GDHt was found to be six times higher than that in *C. butyricum* (2.37 U/mL), and its specific activity was 36.3 U/mg [70], suggesting that protein engineering can be used as a ideal research direction to improve the enzyme activity (Fig. 2).

**Fig. 3:**
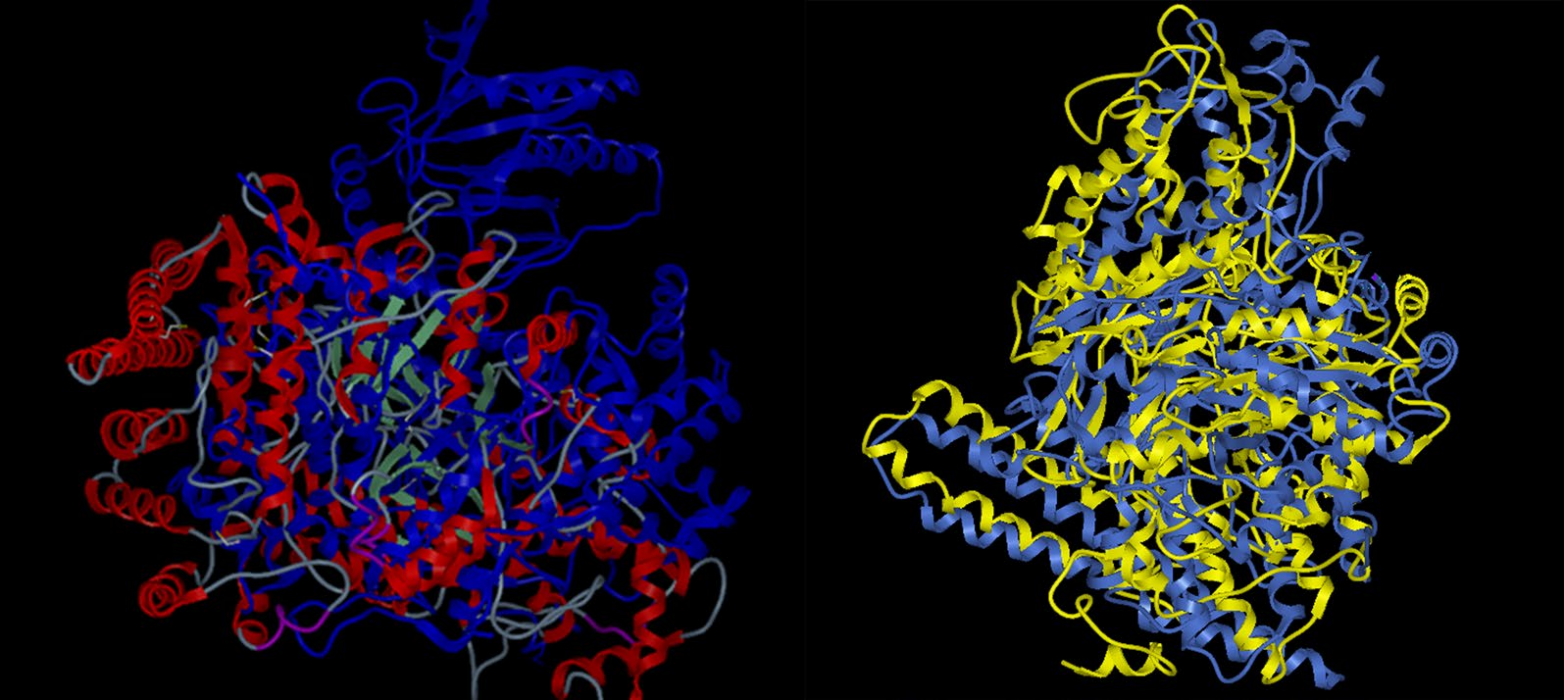
The overlapping of tertiary structures between B_12_-dependent GDHt and B_12_-independent GDHt (*left*) and between B_12_-independent GDHt and PFL (*right*)

The software Molsoft ICM-Pro was used to maximize the overlap of coenzyme-dependent and -independent GDHt tertiary structures, and the result is shown in Fig. 3 (left). Although both were dimers, they showed a vast difference in spatial structures and less overlap. The root mean square deviation (RMSD) was introduced as a parameter to measure the overlapping effect. The greater the RMSD, the lesser the overlap. The overlapping RMSD was 27.104749. On comparing the overlap from *C. novyi* persistent functional language (PFL) and independent GDHt, the similarity was found to be 78 %, and the RMSD was found to be 9.607466 (right), as shown in Fig. 3.

It has been demonstrated that the C-terminal-conserved domain is the binding site of the GDHt and its reactivators. Coenzyme B_12_-independent GDHt is a monomer-subunit dimer [71]. Two monomers, which constitute the GDHt dimers through a noncovalent bond and comprise 10 β/α barrel structure, had almost perfect centrosymmetry. The C terminus is a highly conservative region and a reactivator-binding site. The β/α barrel structure, the substrate glycerol, or 1,2-propylene-binding region of the GDHt is similar to the β/α barrel structure of the PFL and anaerobic ribonucleotide reductase. The most conservative regions in the *C terminus* of the GDHt and PFL are the corresponding amino acid residues 731–782 of the former and 702–754 of the latter, respectively. Moreover, the RMSD is only 7 nm. Using site-directed mutagenesis, O’Brien et al. had demonstrated that R782 residues participated in the proton transfer in the enzyme catalytic process [72]; hence, the C-terminal conserved domain is identified as the binding site of the GDHt and its reactivators. However, the research about the bonding mechanism is scarce.

As all B_12_-dependent GDHts need a number of coenzymes, vitamin B_12_ leads to the high cost of biological process for producing 1,3-PD. It would be an ideal research direction to modify the B_12_-dependent GDHt mutate to the B_12_-independent GDHt (Fig. 2). Moreover, the coenzyme B_12_-independent GDHt should help to improve the development of an economic and vitamin B_12_-free procedure for conversion of renewable resources such as glucose to 1,3-PD.

### Cloning and characterization of the GDHt

Macis et al. first reported the sequences of genes encoding key enzymes (GDHt) involved in glycerol bioconversion to 1,3-PD [51]. Then, the genes from several bacteria, *C. freundii*, *Cl. pasteurianum*, *C. Butyricum*, and *K. pneumoniae*, encoding GDHt were cloned and characterized [33, 51, 58, 59]. Names and lengths of genes, protein molecular masses of GDHt and DDH from different organisms are shown in Table 1. Yakusheva et al. [73] made a sensitive new recording method for determining the activity of GDHt by modifying the two calorimetric methods elaborated [74]. Ahrens et al. modified this method [50] and used a correction factor of 1.41. With different host bacteria, the activity of the GDHt was different, which was shown by Macis et al. [51]. Even through there are a large number of reports about the cloning and characterization (Fig. 2) of the B_12_-dependent GDHt, the characterization of coenzyme B_12_-independent GDHt is poorly understood.

**Table 1:** The codings of GDHt and DDH from different organisms

Organism	Gene name			Gene length			Protein molecular mass (Da)		
	α	β	γ	α	β	γ	α	β	γ
*K. pneumoniae*	*gldA*	*gldB*	*gldC*	1668	585	426	60,621	21,310	16,094
*K. oxytoca*	*pddA*	*pddB*	*pddC*	1668	675	522	60,348	24,113	19,173
*K. pneumoniae* XJPD-Li	*dhaB*	*dhaC*	*dhaE*	1668	584	425	60,702	21,322	16,101
*C. pasteurianum*	*dhaB*	*dhaC*	*dhaE*	1665	540	441	60,813	19,549	16,722
*C. freundii*	*dhaB*	*dhaC*	*dhaE*	1665	540	441	60,813	19,549	16,722
*C. freundii*	*dhaB*	*dhaC*	*dhaE*	1668	585	429	60,433	21,487	16,121
*Clostridium butyrium*	*dhaB1*			2364			88,074		
*S.typhimurium*	*pduC*	*pduD*	*pduE*	1665	675	522	60,307	24,157	19,131

The *dha*BCE genes of *K. pneumoniae* XJPD-Li, *C.pasteurianum* and *C.freundii*; the *gld*ABC genes of *K.pneumoniae* encode coenzyme B_12_-dependent glycerol dehydratase; The *pdd*ABC genes of *K.oxytoca* and the *pdu*CDE genes of *S.typhimurium* encode coenzyme B_12_-dependent diol dehydatase; The *dhaB1* gene of *Clostridium butyrium* encode coenzyme B_12_-independent glycerol dehydratase; α, large subunit; β, intermediate subunit; γ, small subunit

### Bioinformatics and crystal structure of the GDHt

The mechanisms of coenzyme-dependent GDHt and DDH have been studied extensively and are fairly well understood [75, 76]. Crystal structures of the coenzyme-dependent GDHt, which forms a complex with K^+^, cobalamin, and propane-1,2-diol, respectively, were reported [66, 77]. The biological form and the subunit composition of the coenzyme-dependent GDHt are α_2_β_2_γ_2_ heterohexamers [66].

The GDHt is assembled in the form of a dimer of αβγ heterotrimers. Moreover, the α subunit contains a triosephosphate isomerase (TIM) barrel structure with the active site isolated inside the central barrel formed by eight parallel β strands. The α subunit is a coenzyme-dependent activity of GDHt center, which contains essentially factor K^+^-binding sites. Coenzyme B_12_ vitamins are located in between the TIM barrel structure and β subunit. K^+^ ions, the substrate molecule, and coenzyme adenosine bind only with α subunit. The B_12_ is sandwiched between the open end of the central barrel and the β subunit. It was demonstrated that once the GDH-B_12_-K^+^ complex was formed, even with the substrate, the K^+^ ion in the active site is unlikely to readily exchange with other monovalent cations such as NH_4_^+^, while the substrate is likely to reduce the mobility of the K^+^ in the active site [77]. The structure–function relationship of the coenzyme-dependent GDHt with its coenzyme has also been investigated extensively [78–81], and the crystal should be of help in studying the structure–function relationship and performing evolution by genetic engineering (Fig. 2).

### Genetic engineering and protein engineering of the enzyme

As the activity of GDHt is a limiting step in the production of 1,3-PD, especially at high concentrations of glycerol [50, 65], it is suggested that increasing the enzyme activity could contribute to an increase in the productivity of 1,3-PD from renewable resources. At present, there are several challenges to be addressed in the bioproduction of 1,3-PD: when high concentration of glycerol is used, GDHt is the major rate-limiting enzyme in *K. pneumoniae* and *C. butyricum* [50, 65]; high concentrations of glycerol and 3-HPA inhibit GDHt and render irreversible suicide inactivation of GDHt [48, 55, 82, 83]; and the cost of the coenzyme. The improvement in the enzymatic properties of GDHt is desirable for the biosynthesis of 1,3-PD. Genetic engineering and protein engineering (Fig. 2) can improve enzymatic properties and solve these problems for industrial production.

### Rational design

Rational design as a simple and promising method was applied to improve the performance of industrial enzymes such as GDHt. The GDHt is a complex enzyme consisting of three different subunits in the active form of α_2_β_2_γ_2_. Only one study reported about its protein engineering using subunit gene swapping [84]. The thermal, pH stability, and *V*_max_ of the GDHt were markedly improved by 2-5 times compared with the wild type by rational design [84, 85]. PoPMuSiC is an effective computer-aided rational design program for site-mutation study of proteins or polypeptides [86]. Rational design is more efficient, with the sample and the targeting being easier, compared with the directed evolution method (also called irrational design such as error-prone polymerase chain reaction [PCR] and DNA shuffling), and hence, it can be used as a promising method to ameliorate enzymatic properties.

### Saturation-mutagenesis and distant mutations

Saturation-mutagenesis and distant mutations have made a contribution to the toolbox of industrial enzyme amelioration, and there are many successful cases [87–89]. Through these methods, the catalytic activity of a mutant in β-subunit (β-Q42F, 29.6 Å from the active site) was improved by 8.3-fold higher than the wild type, and the catalytic efficiencies of other two mutants β-Q42L and β-Q42S for substrate glycerol were, respectively, 336- and 80-folds higher than that for 1,2-propanediol [90]. The optimal temperature of GDHt, from *K. pneumoniae* XJPD-Li, was 5 °C lower for the mutant compared with the wild type [91]. In comparison, by making a mutant to be near the active sites and the others at distant positions at the same time, some interesting phenomenon might be found.

### Direct expression, coexpression, and fusion expression

Direct expression, coexpression, and fusion expression methods were used for heterologous expression and characterization of GDHt from *K. pneumoniae* in *E. coli*, and the activities of three heterologous expression products were found to be 27.4, 2.3, and 0.2 U/mg, respectively. It was demonstrated that the highest enzyme activity was almost 17 times of that in *K. pneumoniae* [92], while these researches had always focused on only one enzyme. These methods could be used for the coexpressions of *dhaB* and *dhaT* genes to efficiently convert glycerol to 1,3-PD, and even in other bioconversion processes involving multienzymatic coexpressions, such as coexpressions of the GDHt, PDOR, and the reactivating factor.

### Inactivation and reactivation of GDHt

The inactivation of GDHt during catalysis had been demonstrated in many studies [77, 93, 94]. It catalyzes the conversion of glycerol to 3-HPA via a radical mechanism that requires 5′-deoxyadenosylcobalamin (coB_12_) and monovalent cations [77]. Both ammonium and rubidium ions can substitute for K^+^ to activate the enzyme. Moreover, K^+^ is heptacoordinated by the two hydroxyls from the substrate and five oxygen atoms from the active-site residues [66, 95]. Cross-activation had demonstrated that the reactivase from *K. pneumoniae* for GDHt is much slower in reactivating inactive DDH, while the reactivase of *K. oxytoca* for DDH can reactivate at higher rate than that of the inactive GDHt [96]. It has been shown that out of 22 residues, which come from subunit α (16), β (4), and γ (2) identified as potentially important for reactivase specificity, 14 of them have changes in charge between the two groups of dehydratases [94].

In the future, computer modeling, site-directed mutagenesis, and protein engineering could be used to study the reactivase specificity characteristics such as the nature of GDHt/DDHt-reactivase interaction and the GDHt/DDHt interacting surface with the reactivase. It had been demonstrated that the function of GDHt reactivase is to remove damaged coenzyme B_12_ from GDHt, which has suffered mechanism-based inactivation [94]. Based on the structural features, Liao et al. proposed a hypothesis about the reactivation mechanism of reactivase [94]. Both GDHt and its isofunctional homolog DDH undergo mechanism-based inactivation by glycerol during catalysis. This phenomenon was caused by the loss of the intermediate radical from the active site, leaving catalytically incompetent cofactor [cob (II) alamin and 5′-deoxyadenosine] tightly bound in the active site. This would appear to impose a severe limitation to an organism’s ability to ferment glycerol. To overcome this limitation, specific protein-reactivating factors (reactivases) for both GDHt and DDH have been identified, which catalyze the adenosine triphosphate (ATP)-dependent exchange of cob (II)-alamin and 5′-deoxyadenosine for coenzyme B_12_ from the medium [97]. Mori and Toraya et al. have demonstrated that reactivase mediates an ATP-dependent reactivation [98]. It has been proposed and demonstrated that reactivase can be seen as a new type of molecular chaperone involved in the reactivation of the inactivated enzyme [94, 98–100]. The amino acid sequences of the β subunit of the GDHt/DDH and the β subunit of GDHt/DDH reactivases are very similar [100]. The GDHt reactivases are capable of binding to B_12_ from *C. freundii* [48], but there are no reports about reactivases with specific binding activity to cobalamin. The reactivation mechanism had been demonstrated through implications of reactivase structural features. Moreover, the function of reactivase is to help in some other manner with the relaxation of the GDH β-subunit to facilitate release of the damaged cofactor [94].

Reactivating factors for GDHt and DDH were discovered, and the mechanisms of their actions were established [98]. The *gdrA* and *gdrB*, two open-reading frames of *K. pneumoniae*, as the genes for a reactivating factor for GDHt were obtained [101]. The reactivating factors, DdrA and DdrB proteins, are highly homologous to the products of *gdrA* (*dhaB4*) and *gdrB* (*orf2b*) of *K. pneumoniae*, *orfZ* of *C. freundii*, [58] and *orfXC. pasteurianum* [51]. Moreover, the DdrB protein is homologous to the β subunits of DDH [102] and GDHt [58], while the gene product of the orfZ in *K. oxytoca* is considered to be involved in the reactivation of glycerol-inactivated DDH or the enzyme-cyanocobalamin complex [100]. Moreover, the orfZ does not encode a subunit which is required for the activity of GDHt, as in the absence of the orfZ for *C. freundii*, *C. pasteurianum*, *K. oxytoca*, and *K. pneumoniae*, the activity of the enzyme was not lost [51, 58, 59, 102].

**Fig. 4:**
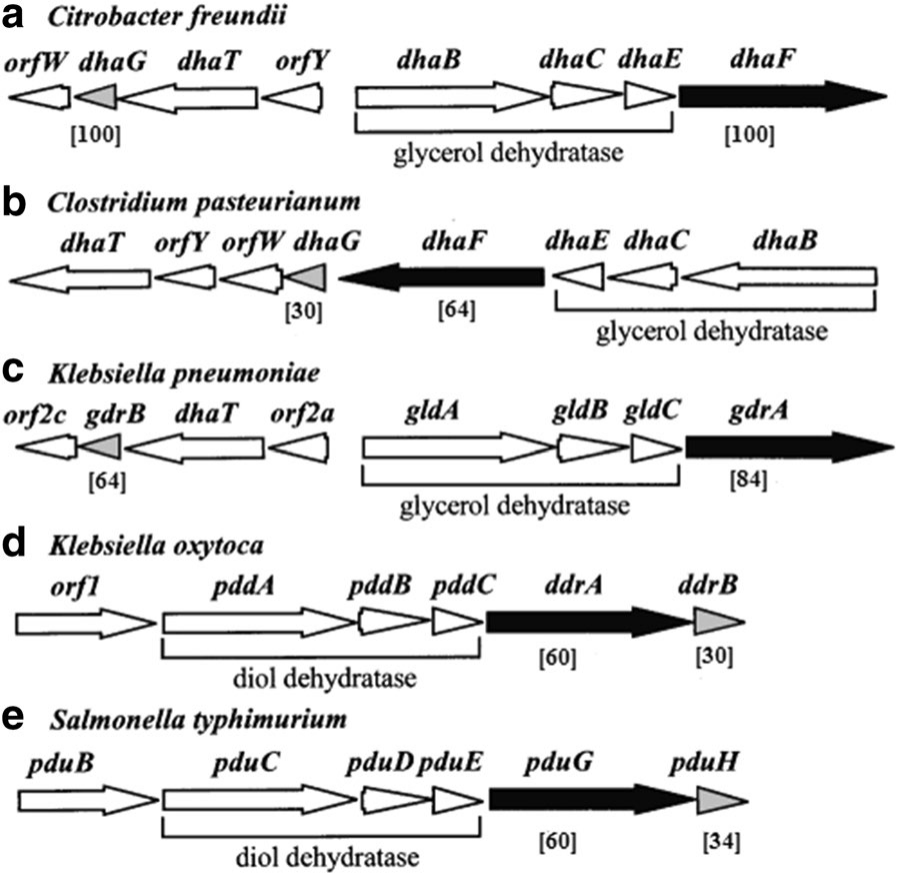
Genes encoding GDHt reactivase and DDH reactivase from diverse microorganisms

GDHt reactivation enzymes are different for four polymers, which comprise four subunits of α_2_β_2_ formed by noncovalent bond hydrophobic interaction [94]. GDHt reactivation has two large partner families: structural features of Hsp70 and GroEL, which are new types of molecular chaperones. The distribution of the genes, which encode GDHt reactivation and 1,2-propylene glycol dehydration reactivation α and β in different strains, is shown in Fig. 4. The different distributions of the GDHt genes give us an option to choose a different method to solve the problems of the enzyme inactivation.

### Enzyme-coupling catalysis

#### GDHt and its activation factor

As GDHt becomes inactivated in the catalytic process in a short time, scholars’ researches were focused more on the activation factor. The irreversible cleavage of the Co–C bond of coenzyme vitamin B_12_, forming 5′-deoxyadenosine and a cobalamin-like species, the mechanism-based inactivation was demonstrated and summarized [76, 96, 103]. Holoenzyme GDHt was also inactivated by O_2_, not by the substrate. This phenomenon is due to the reaction of the coenzyme Co–C bond activation with O_2_ [104]. With free AdoCbI, Mg^2+^, and ATP, the reactivating factor can reactivate glycerol-inactivated, O_2_-inactivated holoenzymes, and activate the enzyme, CN-CbI complex [75, 76]. Cloning, identification, expression, and analysis of the GDHt and its reactivating factor have been reported [48, 59, 83, 105]. The system, which made the inactivated GDHt effectively reactivated, is as follows: the concentrations of coenzyme B_12_, Mg^2+^, and ATP were 3 μM, 10, and 50 mM, respectively, when the ratio (W/W) of reactivation to GDHt factor was 1:4. As a result, the O_2_-inactivated and glycerol-inactivated dehydratase could be reactivated to 97.3 and 98.9 % of initial activity in 10 min, respectively. A reactivating system, with ATP, Mg^2+^ ions, and AdoCbl, has demonstrated that the cobalamin is released from the inactivated enzyme in exchange for free AdoCbl. Moreover, this has been found to occur in GDHt, DDH, and ethanolamine ammonia lyase [106]. Coenzyme B_12_-dependent enzymes may bind to their cofactors in two different ways: the “base-on” mode and the “base-off” mode, which were defined as the reactivation factors together with ATP [107]. It was confirmed that both GDHt and DDH reactions utilize the “base-off” mode of coenzyme-B_12_ [108]. Even though there are many researches about enzyme activation, it is far from achieving practical production, and there is no report on actual catalytic activity.

In our group, two incompatible plasmids, pET-32*gld*ABC and pET-28*gdr*AB, under screening pressure by ampicillin and kanamycin, were used for coexpression of GDHt and its reactivase. The *gld*ABC and *gdr*AB, from *K. pneumoniae* DSM2026, encoded GDHt and its reactivase, respectively. The glycerol-inactivated GDHt in permeabilized cells underwent rapid reactivation in the presence of Mg^2+^, ATP, and coenzyme B_12_ and produced 3-HPA two times more than that without reactivation. This result identified the reactivational ability of GDHt reactivase, and successfully coexpressed GDHt, and its reactivase in vivo with two incompatible recombinant plasmids. This is the first case about using incompatible recombinant plasmids to coexpress GDHt and its reactivase.

#### GDHt and NADH/NAD^+^

While metabolic engineering studies have focused on manipulating enzyme levels using amplification, addition or deletion of a particular pathway, cofactor operations can potentially become a powerful metabolic engineering tool [109]. Cofactors NADH/NAD^+^ play an important role in microbe catabolism. Moreover, for further increase in system productivity, the cofactor manipulation may become crucial.

The natural fermentation process of transformation from glycerol to 1,3-PD and recent developments related to the effort of the metabolic engineering of the novel d-glucose pathway to 1,3-PD have been reviewed [110, 111]. A series of patents and applications regarding the strategy and progress in design and building a single organism catalyst for the direct conversion of d-glucose to 1,3-PD have been reported [112–114]. The equations, which illustrate that this process has a redox balance, of the chemical transformation of glycerol (Eq. 1) versus d-glucose to 1,3-PD (Eq. 2), are as follows: [110].

1 $${\text{Glycerol + }}{\text{NADH + H}}^{ + } \to 1,{ 3} - {\text{Propanediol }} + {\text{ NAD}}^{ + } + {\text{H}}_{ 2} {\text{O}}$$

2 $$\begin{aligned} 1/ 2_{\text{D}}\text{-}{\text{Glucose }} + &\sim {\text{P}}_{\text{i}} + {\text{ 2NADH }} + {\text{ 2H}}^{ + } \\ &\to 1,{ 3}\text{-}{\text{Propanediol }} + {\text{ 2NAD}}^{ + } + {\text{ P}}_{\text{i}} + {\text{ H}}_{ 2} {\text{O}} \\ \end{aligned}$$

Berríos-Rivera et al. demonstrated that it is possible to increase the availability of intracellular NADH through metabolic engineering to provide a more reduced environment both under anaerobic and aerobic conditions [109].

#### *dhaB1* and *dhaB2* YqhD

The genes, *dhaT* and *dhaB*, from *C. freundii* or *K. pneumoniae* were also used to produce 1,3-PD by coupling catalysis in *E. coli* [110], through coexpression of the *dhaB1* and *dhaB2* genes, which encode the vitamin B_12_-independent GDHt, DhaB1, and its activating factor, DhaB2, respectively. The *yqhD* gene encodes the PDOR isoenzyme *dhaT*, an NADP-dependent dehydrogenase. Moreover, it can directly convert glycerol to 1,3-PD, and the highest yield and the efficiency of productivity of 1,3-PD were obtained compared with other reports [115]. The method has further promise for industrial applications, as it overcomes the consumption of isopropyl β-D-1-thiogalactopyranoside and helps in enlarging the substrate range of the 1,3-PD synthesis pathway to more-abundant renewable feedstock such as sugar and starch.

#### GDHt and PDOR

As PDOR catalyzes the reaction that converts 3-HPA to 1,3-PD, and a high concentration of 3-HPA makes the GDHt to lose its activity. The enzyme-coupling catalysis of PDOR and GDHt probably improves the accumulation of 1,3-PD. Also the GDHt, the reactivating factor, and PDOR can be coexpressed in one host to construct an engineered bacterium, which could greatly improve the efficiency of converting glycerol to 1,3-PD.

The effects on glycerol fermentation of GDHt overexpressing separately or collaboratively with PDOR in *K. pneumoniae* DSM2026 were investigated, and the role of DhaR as a positive regulator of the *dhaT* gene had been demonstrated [21]. The enzymes GDHt and PDOR obtained from *K. pneumoniae* have been overexpressed in *E. coli* [47, 116]. Zhang and Jian established a temperature-controlled expression vector pHsh harboring y*qh*D, *dha*B, *dha*G, and *dha*F and produced an increase in the recombinant strain yield of 1,3-PD by 28 % [117]. Huijin et al., Zheng et al., and Zhu et al. used coexpressing GDHt and PDOR or PDOR isoenzymes to produce 1,3-PD [21, 118, 119]. The US patent is based on a recombinant bacterium, which contains genes encoding GDHt, PDOR, and vitamin B_12_ transport proteins, connecting ATP, vitamin receptor precursor gene, and vitamin B_12_ transfer protein gene. Moreover, the recombinant bacterium can undergo fermentation to produce 1,3-PD [120]. In our group, the recombinant plasmids pET-32a-*gld*ABC (for cellular expression) and pET-22b-*gld*ABC (for extracellular expression) have been constructed [70]. The enzymes have been found to be 0.476 and 0.224 U/mL and have six and three times of activities of the wild-type strain, respectively [70]. Through the introduction of two restricted enzyme positions between *gld*A and *gld*C genes, directed evolution of mutant library of the *gldB* was built to improve the enzymatic properties using error-prone PCR [121].

#### GDHt, O_2_, and NADH oxidase

With O_2_ as the substrate, NADH oxidase can produce H_2_O. It is ideal to build the coupling system that coexpresses NADH oxidase with the coenzyme B_12_-independent GDHt, which is sensitive to oxygen. As the B_12_-independent GDHt does not need coenzyme vitamin B_12_ and avoids a biological process with high cost for producing 1,3-PD, it should help in the development of an economic B_12_-independent process for the production 1,3-PD from renewable resources.

### Application of GDHt in the bioconversion of glycerol to 1,3-PD

The biotechnological production of 1,3-PD from glycerol has been demonstrated for several bacteria such as *Lactobacillus brevis*, *Lactobacillus buchnerii*, *Bacillus welchii*, *C. freundii*, *K. pneumoniae*, *C. pasteurianum*, *C. butyricum*, and *E. agglomerans* [19, 29, 55, 64, 122–127]. The amount of 1,3-PD can be increased greatly, while these original strains are reformed for genetic improvement.

So far, there have been two alternatives for manufacturing 1,3-PD using biotechnological route: using natural bacterial strains such as *C. freundii*, *K. pneumoniae*, and *C. butylricum*; and utilizing glucose as feed stock by employing recombinant bacteria. Coexpressing GDHt and dehydratase reactivation factor in recombinant *E. coli* offer a promising potential in enhancing the ability of producing glycerol (from glucose) [128]. Using an alternative substrate such as glucose instead of glycerol could be cost effective in the production of 1,3-PD for making a ‘‘green’’ polyester. 1,3-PD may be the first bulk chemical produced by engineering a bacterium by combining the pathway from glucose to glycerol successfully with a genetically engineered microorganism. Glycerol/glucose-fed batch cofermentations of *E. coli* AG1 harboring these synthetic operons, which were constructed using *dhaB* and *dhaT* under the control of a single trc promoter by Skraly et al., yielded a final concentration of 6.33 g/L 1,3-PD [116]. Nevoigt and Stahl demonstrated the expressions of *dha B*,*C*,*E* and *dhaT* in prokaryotes and eukaryotes [129], but 1,3-PD was detected at very low concentrations. The highest yield and productivity of 1,3-PD were 104.4 g/L and 2.61 g/L/h, respectively, while using glycerol as the sole carbon source. They were obtained by engineering a novel polycistronic operon under the control of the temperature-sensitive lambda phage P_L_P_R_ promoter in *E.coli*, and the rate of bioconversion of glycerol to 1,3-PD attained 90.2 % (g/g) [115].

The main research direction, research content, and possible future directions of the GDHt are summarized in Fig. 2. The train of thought and framework of the GDHt can provide reference for the study of other enzymes, and these conclusions also help to improve the output of 3-HPA and 1,3-PD. These factors (high or low activity of GDHt, high or low sensitivity to the inactivation of the substrate glycerol, its dependence on vitamin B_12_, and its rate of production of 3-HPA), are key in influencing the rate of production of 1,3-PD.

## 1,3-Propanediol oxidoreductase

**Fig. 5:**
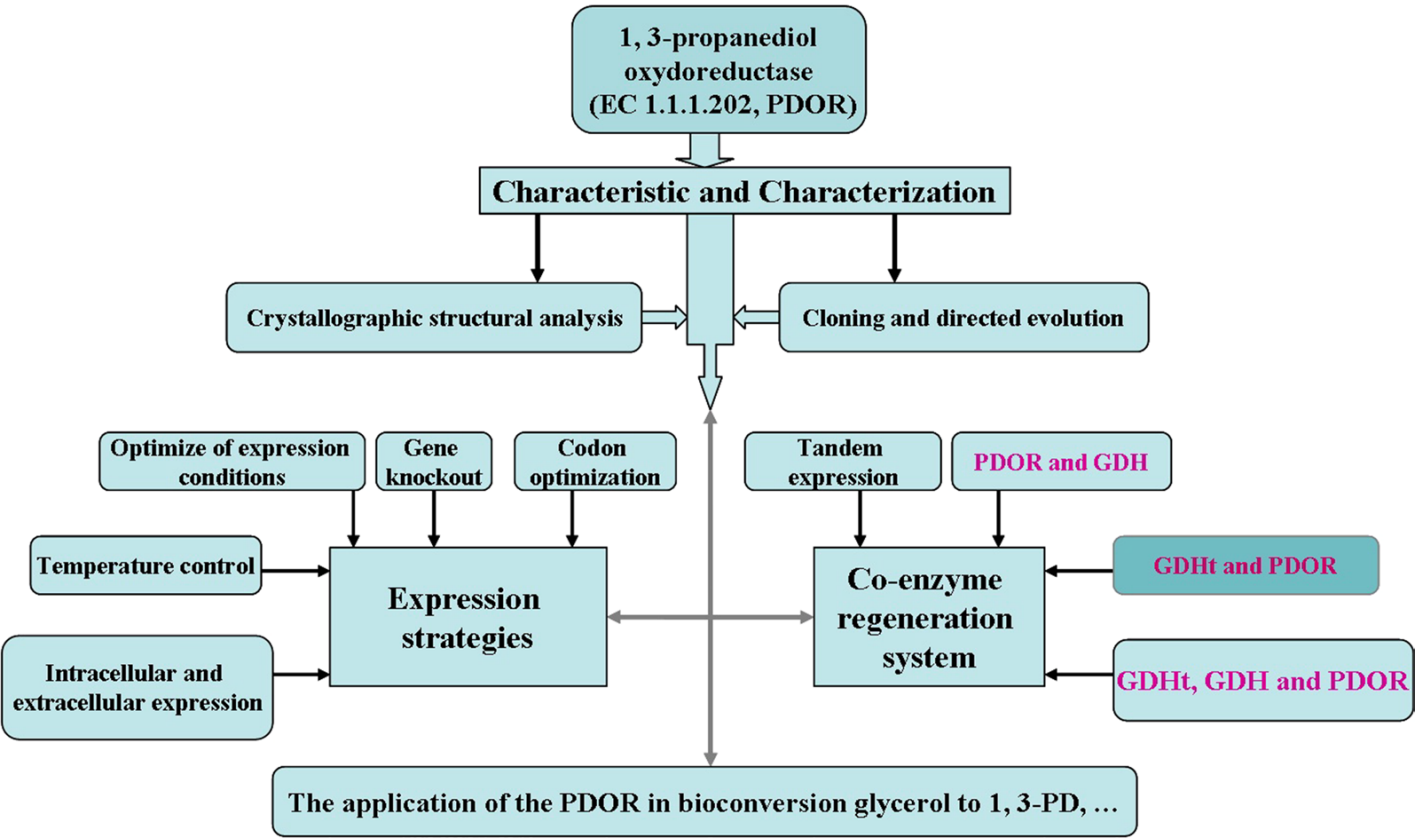
The main research direction about PDOR

PDOR, which directly hydrogenates 3-HPA to 1,3-PD and encoded by *dha*T gene, is one of the key enzymes in the pathway for the conversion of glycerol to 1,3-PD. The PDOR is inhibited by 1,3-PD in metabolic pathways, and hence it resulted in the accumulation of 3-HPA [130]. The accumulation of 3-HPA can inhibit the activity of GDHt to prevent the growth of bacteria and result in reducing the production of 1,3-PD, leading to a major influence in the production of 1,3-PD. Hence, the high or low PDOR activity and the ability of tolerance level of the 1,3-PD are the key factors influencing the concentration of the product of the metabolic pathway. Hence, the importance of study on PDOR is suggested. The main research direction and research content of PDOR are shown in Fig. 5.

### Characteristic and characterization

PDOR belongs to the Fe-NAD-dependent alcohol dehydrogenase third family, and it is also a typical iron-ion activation-type dehydrogenase. In the reduction reaction, PDOR needs NADH as a cofactor that was generated during the oxidation of glycerol to DHA, resulting in the reduction of 3-HPA to 1,3-PD. Moreover, the NADH was oxidized to NAD^+^ at the same time. It needs NAD^+^ as a cofactor in the oxidation reaction.

The 1,3-PD is a natural substrate for PDOR, and the catalytic process needs NAD^+^ as a cofactor. It was demonstrated that the enzyme could also use the 1,4-butanediol, 1-butyl alcohol, 1-propanol, glycerol, or 1, 2-propylene glycol, etc. as the substrate. For the PDORs, from *C. butyricum* E5 and *C. freundii* DSM 30040, the optimal substrate was 3-HPA in the catalytic reduction reaction, and the optimal substrate was 1,3-PD in the catalytic oxidation reaction [55, 131].

Optimal pH for different PDORs showed almost no difference, but the difference varies according to the role. When as a reductase, the optimal pH is about 9.0 [53, 131, 132]. While as an oxidase, the optimal pH is about 6.6 [53, 133]. The optimal temperature for the enzyme was different in different species ranging from 25 to 57 °C. The PDORs contained 380–400 amino acid residues, which usually exist in the form of eight polymers in nature, as well as in the form of four polymers.

The PDOR can be activated by divalent metal ions, but a direct activator for the enzyme has not been reported. Table 2 shows the influence of metal ions on the PDOR enzyme activity. It was reported that the PDOR from *K. pneumoniae* could be reactivated by Mn^2+^ and Fe^2+^ [132]. Mn^2+^ and Fe^2+^ had the activation effect on the PDOR from *C. freundii* DSM 30040 [55]. The PDOR from *C. butyricum* E5 showed that the highest enzyme activity exists only in Mn^2+^, while the enzyme activity declined by 60–90 % in other cations [131]. These results could be considered as effective reference for practical production.

**Table 2:** The effects of PDORs from different organisms by metal ions

Metal ions	Strains	Mark	The data source
Ca^2+^	*Lactobacillus brevis, Lactobacillus buchneri*	–	Veiga-da-Cunha and Foster [53]
Fe^2+^	*Lactobacillus brevis, Lactobacillus buchneri, Citrobacter freundii*	–	Veiga-da-Cunha and Foster MA [53], Daniel et al. [55]
K^+^	*Lactobacillus reuteri*	Highest levels of activity in the presence of 100 mM K^+^	Talarico et al. [133]
Li^+^	*Clostridium butyricum*	–	Malaoui and Marczak [131]
Mg^2+^	*Lactobacillus brevis, Lactobacillus buchneri*	–	Veiga-da-Cunha and Foster [53]
Mn^2+^	*Clostridium butyricum, Lactobacillus brevis, Lactobacillus buchneri, Citrobacter freundii*	–	Veiga-da-Cunha and Foster [53], Daniel et al. [55], Malaoui and Marczak [131]
Na^+^	*Clostridium butyricum*	–	Malaoui, Marczak [131]

### Cloning and directed evolution

**Fig. 6:**
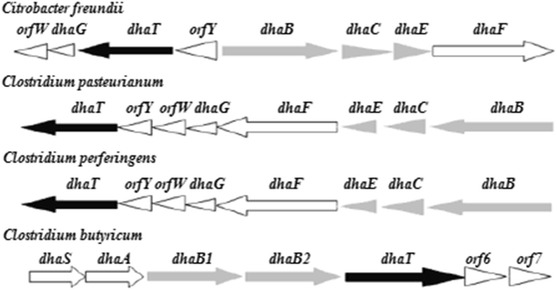
The structure of dha subsystem *Black arrows* indicate a 1,3 propanediol dehydrogenase gene transcription direction

In 1995, for the first time, the *dha*T gene, encoding PDOR, was cloned from *C. freundii*, and it was expressed in *E. coli* [55]. Later, the PDORs were purified from *L. brevis*, *K. pneumoniae*, and *C. butyricum*, and the properties of different microbe PDORs are shown in Table 3. Genes, belonging to the 1,3-PD production strain, encoding PDOR is referred to as the regulation of dha subsystem (Fig. 6). Moreover, the PDOR was encoded by the *dha*T in *C. freundii* and *C. butyricum* [134]. Using a single-factor and uniform design, the most optimal PCR system was built to amplify the *dha*T gene [135, 136]. The *yqh*D, which encodes the PDOR isozyme, was first cloned from *E. coli* by Zhang et al. [117], and the Dupont company is currently using *yqh*D genes to construct genetically engineered bacteria with relatively high performance.

**Table 3:** The properties of PDORs from different microbial sources

Strains	Relative molecular mass (kD)	Subunit relative molecular mass (kD)	Specific activity (U/mg)	*Km* (mmol/L)	Optimal temperature ( °C)	Optimal pH	Metal ions with the enzyme activity
*L. brevis*	350	41–46	7.28	300	37	6.6	Fe^2+^, Mn^2+^: the enzyme activation factors
*K. pneumoniae*	336	42	37	18	30	9.5	
*C. freundii*	347	43.4	11	140	37	7.7	Fe^2+^ or Mn^2+^ can make its recovery activities
*E. agglomerans*	355 ± 5	38.5 ± 0.5	3.42	13.7	37	7.8	Mn^2+^: the enzyme activation factors
*K. pneumoniae* DSM2026	387	41.5	9.85	–	55	10.0	Fe^2+^, Na^+^, NH^4+^, and Mn^2+^: auxo-action on the enzyme activity
*C. butyricum*	384.2 ± 31.1	42	4.51	0.17	37	9.07	Li^2+^, Mn^2+^, and Na^+^: the enzyme activation factors

Enzyme needs to be kept highly active in extreme environments for a long time during practical application. In spite of the effort put into the biological production of 1,3-PD, PDOR is not available commercially [52]. The importance of modifying the natural PDOR is suggested. Moreover, for the research direction and method of modifying the PDOR, one can refer to the research direction of the GDHt (Fig. 2). Error-prone PCR, DNA shuffling, exon shuffling, random priming in vitro recombination, stagger extension process, random insertional–deletional strand exchange mutagenesis, and RAISE are the most common methods of directed evolution. Error-prone PCR is one of the most widely evolutionary means for its simple operation and effective advantages.

PDOR’s activity and its tolerance of 1,3-PD can be improved by at least four methods: (1) using genetic engineering, the gene encoding PDOR was cloned in *E. coli* generating genetically engineered bacteria, and the highly active and highly expressing PDORs were obtained by optimizing the induced conditions; (2) using directed evolution methods (such as error-prone PCR, DNA rearrangement, the family of DNA rearrangement methods) to modify the PDOR molecules, to obtain a PDOR with high activity and resistance to high concentrations of 1,3-PD; (3) combining (1) and (2) to improve the activity of PDOR and its tolerance of 1,3-PD; and (4) changing the mode of production such as enzyme-coupling method which can be used in vitro for 1,3-PD enzymatic production. Some researchers have already started the investigations on these lines [137].

### Crystallographic structural analysis

The PDOR monomer can be categorized into two-functional domain structures: N-terminal domain structure is responsible for the identification and combination of substrates, and C-side domain structure is responsible for the combination of iron ions. Members of the Fe-NAD-dependent alcohol dehydrogenase third family share an iron ion combination model, and this model consists of three histidines (His267, His281, and His202) and an aspartic acid (Asp198). Overall, 387 amino acids comprising monomers formed 13 α helix and 8 β folding. The PDOR monomers, which are similar to any other member of ethanol dehydrogenase third family structure, were categorized into two domains using a hydrophobic pocket. Iron ion combines with the bottom of the pocket and the sites, His267 His281, His202, and Asp198, while the fifth site was set aside to combine with the substrate. The NADH, which is involved in the enzyme reaction, was also predicted to be in the hydrophobic pocket position. The amino acid composition is highly conservative due to the important role of hydrophobic pockets. Moreover, the PDOR crystal structure was parsed for the first time by Rondon et al. [138]. The quaternary structure of PDOR from *K. pneumoniae* (shown in 2009) consists of two closely connected dimer small subunits [138].

Studies on the crystal structure and on the interpretation of its structure would lay a solid foundation to our further understanding of the enzyme molecule catalytic mechanism. Sulzenbacher et al. first parsed the crystal structure of PDOR isoenzyme, which was encoded by *yqh*D [131]. The enzyme comprises two monomer dimers which were similar to the PDOR of *Thermotoga maritima*. Each monomer comprised 387 peptide residues containing two domain structures constituted by Rossmann fold and α helix, and the structural domain contains NADP coenzyme-binding sites [139].

The width of cleft prone changed at various pH conditions. The width of the cleft was relatively small in alkaline environment but large in neutral environment. At the same time, PDOR binds better to the NAD when the cleft narrows. The docking research matches the result, that is, the PDOR’s activities at various pH values can be related to the width of its cleft, and the PDOR has a better activity when it has a narrow cleft [140].

### Expression strategies

In recent years, many expression strategies, optimization of expression conditions, medium components, and conditions of fermentation, codon optimization, intracellular and extracellular expression technology, expression of temperature control, gene knockout etc., were used for the efficient expression of PDOR. Moreover, these expression methods, which are summarized in Fig. 5, also can be used to improve the expression levels of the GDHt, GDH, and other enzymes. The optimization of expression conditions of PDOR was obtained using uniform design and regression analysis [141], but it was not executed in 1,3-PD production. Single-factor design and response surface methodology were used to optimize the medium components and the optimal condition of fermentation for the expression of PDOR [142]. The PDOR isozyme activity increased 4.6 times, while the *yqh*D gene was amplified in the *K. pneumoniae* mutant strains AK, which knocked out the GDH and PDOR [143]. The *dha*D gene encoding GDH and the *dha*K gene encoding dhaK were knocked out to eliminate oxidation to enhance reduction, resulting in an increase in the yield of the 1,3-PD by 2.06 times compared to that of the wild-type *K. pneumoniae* [144]. Moreover, the activities of the GDHt and PDOR have increased by 6.09 and 6.46 times, respectively [144]. The by-products declined, and the production of 1,3-PD increased by 11.8 %, while the three strains, *K. pneumoniae/pEtac*-*yqhD*, *K. pneumoniae/pEtac*-*dhaT* and *K. pneumoniae/pEtac*-*dhaT*-*tac*-*yqhD* were coexpressed [145]. Nakamura and Whited demonstrated that the *yqh*D gene from *E. coli* encoding PDOR isoenzyme was an obvious improvement compared with the PDOR [110].

To achieve the PDOR intracellular and extracellular expressions, the intracellular expression vector pET-22b-*dha*T and the secretion expression vector pET-22b-*dha*T were constructed [119]. The PDOR isozyme from *E. coli* was efficiently expressed using temperature-controlled expression vector, and the expression level was controlled using temperature [117]. These methods also can be used in the field of the GDHt and GDH, etc.

Currently, almost all reports of the PDOR cloning and expression are heterologous expressions using genetic engineering. However, the biggest problem for the functional protein expression in heterologous hosts is that the protein genes possibly use the rare codon of host cells, so that the exogenous gene expression level is either very low or there is no expression at all. The maximum yield of the PDOR produced by the synthetic DNA was 385 U/mL, which is nearly fivefolds higher than that of the wild type (82 U/mL). These results were obtained after the gene *dha*T from *K. pneumoniae* was *de novo* compounded according to the codon usage of *E. coli*, as well as mRNA secondary structure (unpublished).

### Tandem expression and coenzyme regeneration system

There are at least three types of effective recombinant DNA methods that exist for the strain to directly transform the low-cost substrate to 1,3-PD: (1) transferring the genes, which were needed in the conversion of glycerol to 1,3-PD, to the strain that can use low-cost substrate product glycerol; (2) the genes, which can convert low-cost substrates to glycerol, being transferred to the strains that can use glycerol to produce 1,3-PD; (3) all relevant genes in (1) and (2) being transferred to the strains that neither produce glycerol nor 1,3-PD such as *E. coli*. GDHt and PDOR were essential for the production of glycerol fermentation of 1,3-PD. The strain could use glycerol to produce 1,3-PD, while it has the two enzymes, which was demonstrated by Skraly et al. and Zheng et al. [21, 52]. The coexpressions of the PDOR and GDHt in *K. pneumoniae* result in an increase of molar yield from 50.6 to 64.0 % of 1,3-PD [146]. The *dha*T and *dha*B from *K. pneumoniae* were coexpressed in *E. coli* JM109 and *S. cerevisiae*, respectively, while the activities of the two enzymes in the *S. cerevisiae* were more than six times that of their activities in *E. coli* JM109 [147]. Glucose was directly converted to 1,3-PD by the genetically engineered bacterium, which was obtained through the coexpressions of the *dha*B and *dha*T genes (from 1,3-PD production bacteria), and *dar1* and *gpp2* genes (from glycerol-producing bacteria) [110].

The coenzyme regeneration system, regeneration of coenzymes NAD^+^/NADH multienzyme (GDHt, GDH, and PDOR) system, in vitro continuous catalytic production of 1,3-PD, can effectively reduce the cost of production and obtain high commercial value of 1,3-PD and DHA. About 90 types of oxidoreductases have potential commercial application value [148], and approximately 80 % of these oxidoreductases need NAD and NADH as coenzymes and about 10 % need NADP and NADPH as coenzymes. The prices of coenzymes are relatively expensive. Hence, the mode of production directly adds to the cost of production of coenzyme inevitably leading to higher production costs. The coenzyme NADH which was produced by the GDH reduction of glycerol to DHA solves the problem of coenzyme regeneration when PDOR oxidizes 3-HPA into 1,3-PD. Moreover, the DHA—a type of important chemical intermediate, multifunctional reagent, pharmaceutical intermediate, and antiviral agent [149]—was obtained at the same time. The types of coenzyme regeneration methods, which are summarized in Figs. 2 and 5 for the GDHt and PDOR, respectively, can complement each other for cofactor regeneration.

The biotechnological way of producing 1,3-PD from waste biomass (e.g., crude glycerol) is an attractive alternative to traditional chemical production. By studying the characteristics of the key and speed-limiting enzymes in the production of 1,3-PD, efficient engineered bacteria can be constructed, which can improve the ability of fermentation in the production of 1,3-PD. Moreover, constructing an environment-friendly biological transformation path that can use cheap substrates such as sugar to produce 1,3-PD would help to improve the existing production capacity of 1,3-PD. The simulation enzymes, which are manufactured based on the principle of the role of the enzyme, simulate the enzyme’s active center, and the catalytic mechanism would be helpful for the efficient production of 1,3-PD [150]. Moreover, it would be an ideal method to improve the properties of enzyme or create a new enzyme.

## Glycerol dehydrogenase

**Fig. 7:**
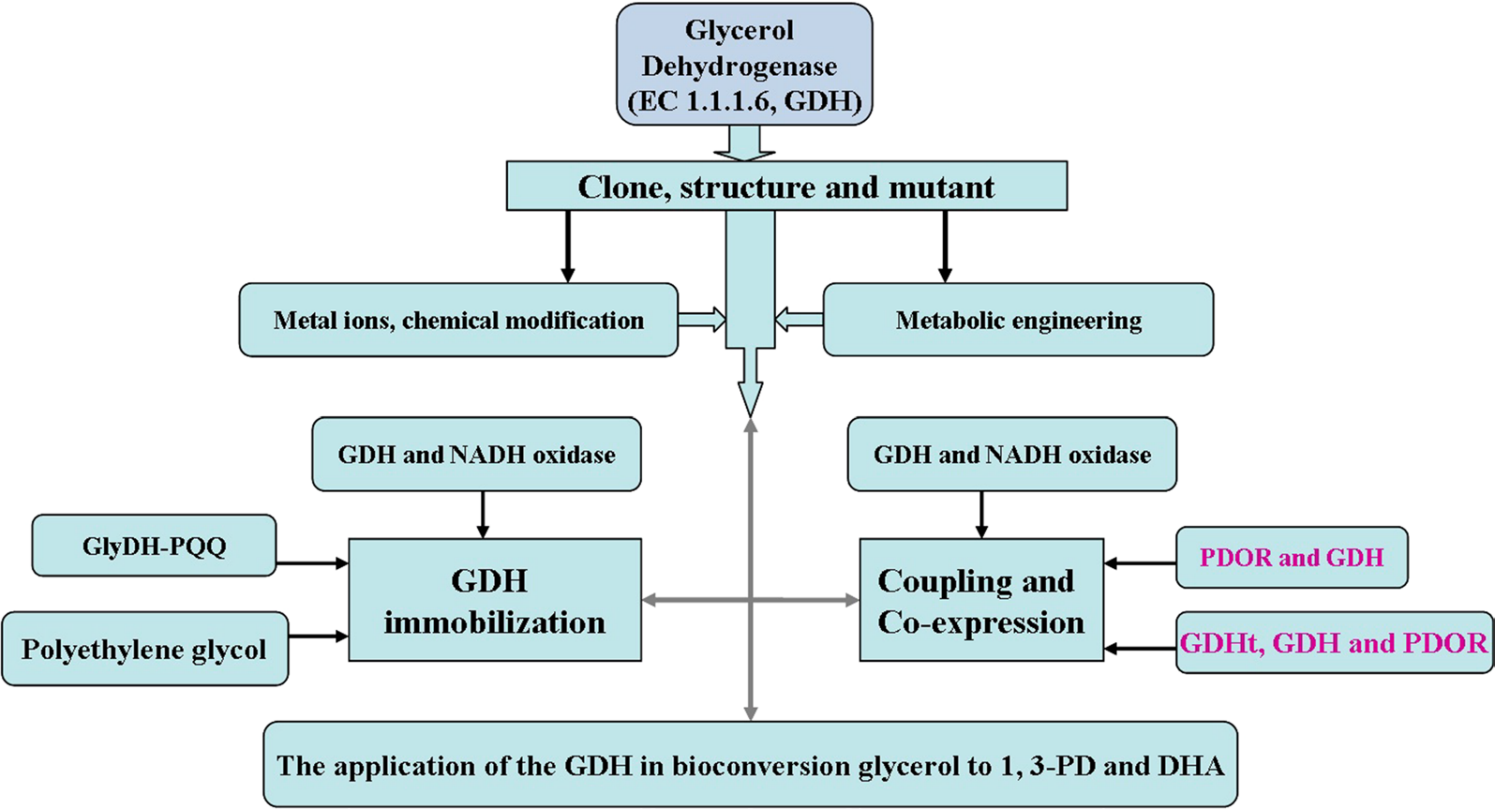
Summary of main research direction of GDHt

GDH is one of the key enzymes in the production of DHA and 1,3-PD by the enzymatic method. The GDHs are categorized into three types depending on the site of oxidation of the glycerol and the nature of the coenzyme required [151]. A summary of main research direction of GDHts is shown in Fig. 7, and there are some directions that are similar to both the GDHt and PDOR (Figs. 2, 5).

### Clone, structure, and mutant

Various GDHs, which originate from *Geotrichum candidum*, *E. coli*, *Zymomonas mobilis*, *Methylotrophic* yeast, *K. aerogenes,* etc., were reported and characterized in recent years. The substrate specificity values of the GDHs from *Hansenula ofunaensis* and *K. pneumoniae*; the kinetic parameters; and the enzymatic properties of GDHs from various organisms are shown in Tables 4, 5, and 6, respectively.

**Table 4:** Substrate specifity values of glycerol dehydrogenase from *Hansenula ofunaensis* and *Klebsiella pneumoniae*

Substrates	Relative activity (%)	
	*Hansenula ofunaensis*	*Klebsiella pneumoniae*
Glycerol	100	100
1,3-Butanediol	160	5
1,2-Propanediol	140	60
1,3-Propanediol	4	2

**Table 5:** Kinetic parameters of GDH from various organisms

Strain	*K*_*m*_	
	Glycerol	NAD^+^
*Schizoaccharomyces pombe*	0.5	0.13
*Klebsiella pneumoniae*	0.75	120
*Citrobacter freundii*	1.27	0.057
*Cellulomonas* sp. NT3060	10.9	0.09
*Clostridium butyricum E5*	91.7	4.07

Units for *Km* (glycerol) is mmol/L, units for *Km* (NAD^+^) is μmol/L

**Table 6:** Enzymatic properties of GDH from various organisms

Enzymatic properties	GDH from *K. pneumoniae* DSM 2026	GDH from *E.coli* BL21(DE3)/pET-*gld*A	GDH from *E.coli* BL21(DE3)/pET-*gld*A-74
Optimal pH	11.0	11.0	12.5
Optimal temperature (°C)	50	65	55
*Km*_(glycerol)_ (mmol/L)	0.79	0.58	0.63
*Km*_(NAD_) (mmol/L)	0.13	0.74	0.77

Richter et al. described the complex functional structures of other members of family 11 of the AKR superfamily. The structure of AKR11B4 shows that the AKR-typical (β/α) 8 TIM-barrel fold, with three loops and the C-terminal tail determining the particular enzymatic properties [151–153]. The crystal and three-dimensional structures of GDHs, which come from different strains or combine with other perssads, are exhibited elsewhere [154, 155]. The GDH encoded by *gldA* gene from *S. marcescens* H30 was expressed in DE3 [22], and the potential application of 2,3-butanediol indicates its properties. The crystal structure of GDH from *Geobacillus stearothermophilus* has been determined and provides structural insights into the enzyme’s catalytic mechanism and substrate specificity. The first structure of the enterobacterial GDH from *Serratia* was reported [154], which is helpful in the study of the diseases caused by gut microbes. The molecular dynamic simulation of the constructed three-dimensional structure of sldha was performed using Gromacs 4.0.5 [156]. This information from the three-dimensional structure of GDH would be beneficial in improving the GDH’s properties using the genetic engineering methods of the GDHt in the Fig. 2.

For industrial application, enzymes are supposed to be highly active in extreme environments such as high temperature, high pressure, and high salt concentration. Therefore, the modification of the natural GDH is needed. The GDH enzyme gene from *K. pneumoniae* was repeatedly amplified by two sequential error-prone PCR. Moreover, the activity of the gldA-74 mutant was shown to be 2.73-fold higher than that of the parent recombinase [157].

### Metal ions and chemical modification

Chemical modification greatly improved the activity and stability of GDH enzyme, which provides greater space for industrial application of GDH. Chemical modification of the GDH was explored in recent years [158]. Spencer et al. described the studies that examined the structure or structures of the metal-depleted enzyme and proposed a model to explain the complex reactivation process observed [159]. Compared to the original activity of GDH (3.17 U/mL), the activities of GDH modified by Mg^2+^, Ba^2+^, and Mn^2+^ were improved by 14.9-, 11.3-, and 12.4-folds after optimization, respectively [160]. The GDH catalytic zinc ion substitution by other divalent metal ions, Mn^2+^ and Mg^2+^, had increased the activities of GDH; however, their thermostability and catalytic promiscuity have not yet been studied [161] (Table 7). The mechanism of the metal ion’s role in the catalytic enhancement was explained [12]. After OPA modification, the GDH from *E. aerogenes* showed a proximal lysine residue to form thioisoindole derivatives. The reaction catalyzed by the GDH was analyzed, and the results showed that the GDH and alcohol dehydrogenases have arrived at a common catalytic solution in spite of their different active site components [162]. These results indicated that metal ion substitutions could be applied to improve the catalytic properties of GDH.

**Table 7:** Metal ions and chemical modification of the GDH

Source	Metal ions	Activity (fold)	Thermostability
*Klebsiella pneumoniae*	Mg^2+^	14.9	Increased
	Ba^2+^	11.3	–
	Mn^2+^	12.4	Increased

### Metabolic engineering

Metabolic engineering can significantly improve the application of GDH in the industrial production of 1,3-PD and DHA. The ability of glycerol metabolism was significantly improved by coexpressing GDH and dhaK in recombinant *E. coli*, and increased by 42 % compared with the wild strain [163]. The process of simulation design and technoeconomic analysis of production technology of GDH were built by Zeng and his co-workers [164]. Being strong promoters, tufB and gdh, were used to overexpress the film system dehydrogenase *sldAB* gene from *Gluconobacter oxydans*, which increases the density of bacterial growth and significantly increases the concentration and rate of conversion. It was demonstrated that the GDH, encoding *gldB* in *Aspergillus nidulans,* was essential for osmotolerance [165]. The *gld1* gene, which encodes GDH, was de-repressed in scr1 Delta and tup12 Delta strains, and it was regulated by glucose repression [166]. Using AT-rich codons immediately downstream of the initiation codon strengthens the expression efficiency of recombinant proteins, while it did not affect the enzymatic properties of recombinant protein [167], suggesting this method can be used for 1,3-PD production.

Overexpression of GDH could decrease the yield of ethanol and 2,3-butanediol and increase the concentration of acetic acid [168]. When the GDH is overexpressed, the newly developed strain *G. oxydans* with high productivity generated resistance in the process of industrial bioconversion of potential DHA production [169]. Enhanced production of DHA from glycerol was due to the overexpression of GDH in an alcohol dehydrogenase-deficient mutant of *G. oxydans* [169]. Using these methods for producing 1,3-PD should be implemented in the future.

### Immobilization of GDH

The immobilized GDH shows better properties than the free GDH. Immobilization of GDH was studied in recent years [2, 170]. The next step is to find more convenient immobilization methods for GlyDH-PQQ, where the solution might be found by the application of more protein-friendly environments such as sol–gels or conducting polymers [171]. The immobilized GDH showed less sensitivity to pH and temperature changes, and exhibited a 5.3-fold improvement in thermal stability at 50 °C, while it exhibited excellent reusability [172]. However, ten cycles of reuse led only to 9 % loss of enzyme activity. The GDH with higher stability and lower product inhibition was obtained using three recombinant GDHs from *G. stearothermophilus*, *C. braakii*, and *Cellulomonas* sp. Moreover, it was stabilized by fixing these enzymes with polyethylene glycol [173].

### Coupling and coexpression

The GDH was used for the products 1,3-PD and DHA through the methods such as enzyme coupling and coexpression. Zhao et al. illuminated the coexpressions of the GDH and PDOR in *K. pneumoniae* for improving their effects on the conversion of glycerol to 1,3-PD in the resting cell system [168]. A newly developed strain *G. oxydans* M5AM/GDH with high productivity and increased resistance to product inhibition was obtained [169]. The kinetics of the GDH and PDOR of *K. pneumoniae* were studied [174], which is helpful to improve our understanding how these enzymes are regulated and for further enzyme catalysis and metabolic engineering studies.

GDH enzyme’s catalytic glycerol production of DHA needs NAD^+^ as a coenzyme and therefore is expensive. The generated reduced coenzyme NADH is the competitive inhibitor of NAD^+^; hence, it is necessary that the coenzyme is recycled. In recent years, scholars like Baishan Fang have started considering the use of enzyme-coupling reaction [168, 175, 176], a coenzyme regeneration in the process of enzyme catalysis, to solve this problem. The coenzyme regeneration can be achieved by enzyme-coupling system of GDH with NADH oxidase or GDH with PDOR (Figs. 1, 5, 7).

## Conclusions

In general, use of renewable waste substrates is an environment-friendly choice that also contributes to the reduction in the costs of waste treatment and increases the economic value of by-products. Most research studies have concentrated on the use of glycerol in production of solvents such as DHA and 1,3-PD. Expanding the glycerol-based natural process to a more efficient process based on less-expensive carbon feedstock involves several steps: changing the anaerobic process to an aerobic one; replacing the feedstock uptake (transport) mechanism of the host organism; applying intergeneric transfer of complex metabolic pathways; and adopting both the design and implementation of an optimal solution to the balance of carbon, redox, and energy with regard to microbial growth and product formation.

To form a metabolic pathway, coexpressions of the key enzymes such as GDHt, PDOR, GDHt reactivase factor, DDH, etc. could directly convert cheap substrates such as glucose to 1,3-PD, and it would be ideal for the environment and recycling of resource. Some studies have been performed on this line, but it is necessary to ameliorate the metabolic system for improving the rate of conversion and productivity of 1,3-PD. Improving the societal benefit was obtained through the production of chemicals from renewable resources. The success of the project bodes well for future metabolic engineering efforts. While the study of GDHt was mainly concentrated on the coenzyme B_12_-dependent GDHt, the research on coenzyme B_12_-independent GDHt needs to be further explored due to it is increased stability, low cost for reactivation, and there being no need for coenzyme B_12_. It could be helpful for glycerol bioconversion and large-scale production of 1,3-PD by applying collective removal method for different types of impurities and carrying out a feasibility study of its industrial scale application. In addition, the technology of integrating catalysis, genetic engineering, protein engineering, immobilized enzyme, and the preparation of simulated enzymes holds promises for finding more new varieties of GDHt, PDOR, and GDH, and finding new strains that have the new GDHt, PDOR, or GDH (Figs. 2, 5, 7) was useful for promoting the improvement of other enzymes’ properties and the development of an ecofriendly and economic biological process for the industrial production of 1,3-PD or other important chemicals from renewable resources.

Changing the mode of production such as the enzyme-coupling method is used in the in vitro catalytic production of 1,3-PD. Compared to traditional microbial fermentation, the enzyme-coupling method has some advantages: use of enzyme catalysis technology maximizes the yield of the product and production capacity; coenzyme regeneration measures can reduce the high costs of adding coenzyme; the product of 1,3-PD can be separated in time, and thus it is no longer needed to consider the question of inhibition of 1,3-PD and PDOR; making full and optimal use of raw and auxiliary materials realizes “zero discharge” of the production process and enables easier-to-implement cleaner production.

## Abbreviations

1, 3-PD: 1, 3-propanediol
DHA: dihydroxyacetone
GDHt: glycerol dehydratase
PDOR: 1,3-propanediol-oxydoreductase
GDH: glycerol dehydrogenase

## References

[1] da Silva GP, Mack M, Contiero J (2009). Glycerol: a promising and abundant carbon source for industrial microbiology. Biotechnol Adv.

[2] Kumar GS, Wee Y, Lee I, Sun HJ, Zhao X, Xia S, Kim S, Lee J, Wang P, Kim J (2015). Stabilized glycerol dehydrogenase for the conversion of glycerol to dihydroxyacetone. Chem Eng J.

[3] Liu L, Zhuge X, Shin HD, Chen RR, Li J, Du G, Chen J (2015). Improved Production of Propionic Acid in Propionibacterium jensenii via Combinational Overexpression of Glycerol Dehydrogenase and Malate Dehydrogenase from *Klebsiella pneumoniae*. Appl Environ Microbiol.

[4] Cremonez PA, Feroldi M, Nadaleti WC, de Rossi E, Feiden A, de Camargo MP, Cremonez FE, Klajn FF (2015). Biodiesel production in Brazil: current scenario and perspectives. Renewable Sustainable Energy Rev.

[5] López BC, Cerdán LE, Medina AR, López EN, Valverde LM, Peña EH, Moreno PAG, Grima EM (2015). Production of biodiesel from vegetable oil and microalgae by fatty acid extraction and enzymatic esterification. J Biosci Bioeng.

[6] Ferrero GO, Almeida MF, Alvim-Ferraz MC, Dias JM (2015). Glycerol-enriched heterogeneous catalyst for biodiesel production from soybean oil and waste frying oil. Energy Conversion Manage.

[7] Willke T, Vorlop K-D (2004). Industrial bioconversion of renewable resources as an alternative to conventional chemistry. Appl Microbiol Biotechnol.

[8] Demirbas M, Balat M (2006). Recent advances on the production and utilization trends of bio-fuels: a global perspective. Energy Conversion Manage.

[9] Su M-Y, Li Y, Ge X-Z, Tian P-F (2014). Insights into 3-hydroxypropionic acid biosynthesis revealed by overexpressing native glycerol dehydrogenase in *Klebsiella pneumoniae*. Biotechnol Biotechnological Equipment.

[10] Mahadevan A, Gunawardena DA, Karthikeyan R, Fernando S (2015). Potentiometric vs amperometric sensing of glycerol using glycerol dehydrogenase immobilized via layer-by-layer self-assembly. Microchim Acta.

[11] Reed P (2015). Making war work for industry: the United Alkali Company’s central laboratory during world war one. Ambix.

[12] Fang B, Niu J, Ren H, Guo Y, Wang S (2014). Mechanistic Study of Manganese-Substituted Glycerol Dehydrogenase Using a Kinetic and Thermodynamic Analysis. PLoS ONE.

[13] Ho S-H, Wong Y-D (2014). Chang VW-C. Evaluating the potential of biodiesel (via recycled cooking oil) use in Singapore, an urban city. Resour, Conservation Recycling.

[14] Samul D, Leja K, Grajek W (2014). Impurities of crude glycerol and their effect on metabolite production. Ann Microbiol.

[15] Sivasakaran C, Ramanujam PK, rajJoseph Xavier V, Arokiasamy WJ, Mani J: Bioconversion of Crude Glycerol Into Glyceric Acid: A Value Added Product.

[16] Colin T, Bories A, Lavigne C, Moulin G (2001). Effects of acetate and butyrate during glycerol fermentation by *Clostridium butyricum*. Curr Microbiol.

[17] Wilke D (1999). Chemicals from biotechnology: molecular plant genetics will challenge the chemical and the fermentation industry. Appl Microbiol Biotechnol.

[18] Himmi EH, Bories A, Barbirato F (1999). Nutrient requirements for glycerol conversion to 1, 3-propanediol by *Clostridium butyricum*. Bioresour Technol.

[19] Yang G, Tian J, Li J (2007). Fermentation of 1, 3-propanediol by a lactate deficient mutant of *Klebsiella oxytoca* under microaerobic conditions. Appl Microbiol Biotechnol.

[20] Du C, Yan H, Zhang Y, Li Y, Cao Z (2006). Use of oxidoreduction potential as an indicator to regulate 1, 3-propanediol fermentation by *Klebsiella pneumoniae*. Appl Microbiol Biotechnol.

[21] Zheng P, Wereath K, Sun J, van den Heuvel J, Zeng A-P (2006). Overexpression of genes of the dha regulon and its effects on cell growth, glycerol fermentation to 1, 3-propanediol and plasmid stability in *Klebsiella pneumoniae*. Process Biochem.

[22] Zhang L, Xu Q, Peng X, Xu B, Wu Y, Yang Y, Sun S, Hu K, Shen Y (2014). Cloning, expression and characterization of glycerol dehydrogenase involved in 2, 3-butanediol formation in *Serratia marcescens* H30. J Ind Microbiol Biotechnol.

[23] Karve M, Patel JJ, Patel NK (2014). Bioconversion of glycerol. J Critical Rev.

[24] He A, Topolkaraev VA, Wright AE, Wideman GJ (2014). Fibers formed from aromatic polyester and polyether copolymer.

[25] Knietsch A, Bowien S, Whited G, Gottschalk G, Daniel R (2003). Identification and characterization of coenzyme B_12_-dependent glycerol dehydratase-and diol dehydratase-encoding genes from metagenomic DNA libraries derived from enrichment cultures. Appl Env Microbiol.

[26] Herva ME, Zibaee S, Fraser G, Barker RA, Goedert M, Spillantini MG (2014). Anti-amyloid compounds inhibit α-synuclein aggregation induced by protein misfolding cyclic amplification (PMCA). J Biol Chem.

[27] Papanikolaou S, Ruiz-Sanchez P, Pariset B, Blanchard F, Fick M (2000). High production of 1, 3-propanediol from industrial glycerol by a newly isolated *Clostridium butyricum* strain. J Biotechnol.

[28] Koutinas AA, Vlysidis A, Pleissner D, Kopsahelis N, Garcia IL, Kookos IK, Papanikolaou S, Kwan TH, Lin CSK (2014). Valorization of industrial waste and by-product streams via fermentation for the production of chemicals and biopolymers. Chem Soc Rev.

[29] Deckwer WD (1995). Microbial conversion of glycerol to 1, 3-propanediol. FEMS Microbiol Rev.

[30] Nemeth A, Kupcsulik B, Sevella B (2003). 1, 3-Propanediol oxidoreductase production with *Klebsiella pneumoniae* DSM2026. World J Microbiol Biotechnol.

[31] Mu Y, Teng H, Zhang D-J, Wang W, Xiu Z-L (2006). Microbial production of 1, 3-propanediol by *Klebsiella pneumoniae* using crude glycerol from biodiesel preparations. Biotechnol Lett.

[32] Lin R, Liu H, Hao J, Cheng K, Liu D (2005). Enhancement of 1, 3-propanediol production by *Klebsiella pneumoniae* with fumarate addition. Biotechnol Lett.

[33] Raynaud C, Sarçabal P, Meynial-Salles I, Croux C, Soucaille P (2003). Molecular characterization of the 1, 3-propanediol (1, 3-PD) operon of *Clostridium butyricum*. Proc Natl Acad Sci USA.

[34] González-Pajuelo M, Andrade J, Vasconcelos I (2004). Production of 1, 3-propanediol by *Clostridium butyricum* VPI 3266 using a synthetic medium and raw glycerol. J Ind Microbiol Biotech.

[35] González-Pajuelo M, Andrade J, Vasconcelos I (2005). Production of 1, 3-propanediol by *Clostridium butyricum* VPI 3266 in continuous cultures with high yield and productivity. J Ind Microbiol Biotech.

[36] Achilias DS, Bikiaris DN. Synthesis, Properties, and Mathematical Modeling of Biodegradable Aliphatic Polyesters Based on 1, 3-Propanediol and Dicarboxylic Acids. Biodegradable Polyesters. 2015:73–108.

[37] Hao J, Xu F, Liu H, Liu D (2006). Downstream processing of 1, 3-propanediol fermentation broth. J Chem Technol Biotechnol.

[38] Liguori F, Moreno-Marrodan C, Barbaro P (2015). Environmentally friendly synthesis of γ-valerolactone by direct catalytic conversion of renewable sources. ACS Catalysis.

[39] Werkman C, Gillen G (1932). Bacteria producing trimethylene glycol. J Bacteriol.

[40] Zhu MM, Lawman PD, Cameron DC (2002). Improving 1, 3-Propanediol Production from Glycerol in a Metabolically Engineered *Escherichia**coliby* Reducing Accumulation of sn-Glycerol-3-phosphate. Biotechnol Prog.

[41] Gungormusler-Yilmaz M, Cicek N, Levin DB, Azbar N. Cell immobilization for microbial production of 1, 3-propanediol. Critical reviews in biotechnology. 2015(preprint):1–13. 10.3109/07388551.2014.992386 25600463

[42] Biebl H, Menzel K, Zeng A-P, Deckwer W-D (1999). Microbial production of 1, 3-propanediol. Appl Microbiol Biotech.

[43] Toraya T, Honda S, Kuno S, Fukui S (1978). Coenzyme B_12_-dependent diol dehydratase: regulation of apoenzyme synthesis in *Klebsiella pneumoniae* (*Aerobacter aerogenes*) ATCC 8724. J Bacteriol.

[44] Forage RG, Foster MA (1982). Glycerol fermentation in Klebsiella pneumoniae: functions of the coenzyme B_12_-dependent glycerol and diol dehydratases. J Bacteriol.

[45] Maervoet VE, De Mey M, Beauprez J, De Maeseneire S, Soetaert WK (2010). Enhancing the microbial conversion of glycerol to 1, 3-propanediol using metabolic engineering. Organic Process Res Develop.

[46] Toraya T, Kuno S, Fukui S (1980). Distribution of coenzyme B_12_-dependent diol dehydratase and glycerol dehydratase in selected genera of Enterobacteriaceae and Propionibacteriaceae. J Bacteriol.

[47] Tong I-T, Liao HH, Cameron D (1991). 1, 3-Propanediol production by *Escherichia coli* expressing genes from the *Klebsiella pneumoniae* dha regulon. Appl Env Microbiol.

[48] Seifert C, Bowien S, Gottschalk G, Daniel R (2001). Identification and expression of the genes and purification and characterization of the gene products involved in reactivation of coenzyme B_12_-dependent glycerol dehydratase of *Citrobacter freundii*. Eur J Biochem.

[49] Jung WS, Kang JH, Chu HS, Choi IS, Cho KM (2014). Elevated production of 3-hydroxypropionic acid by metabolic engineering of the glycerol metabolism in *Escherichia coli*. Metab Eng.

[50] Ahrens K, Menzel K, Zeng AP, Deckwer WD (1998). Kinetic, dynamic, and pathway studies of glycerol metabolism by *Klebsiella pneumoniae* in anaerobic continuous culture: iII. Enzymes and fluxes of glycerol dissimilation and 1, 3-propanediol formation. Biotechnol Bioeng.

[51] Macis L, Daniel R, Gottschalk G (1998). Properties and sequence of the coenzyme B_12_-dependent glycerol dehydratase of *Clostridium pasteurianum*. FEMS Microbiol Lett.

[52] Skraly FA, Lytle BL, Cameron DC (1998). Construction and characterization of a 1, 3-propanediol operon. Appl Environ Microbiol.

[53] Veiga-Da-Cunha M, Foster MA (1992). 1, 3-Propanediol: nAD^+^ oxidoreductases of *Lactobacillus brevis* and *Lactobacillus buchneri*. Appl Environ Microbiol.

[54] Zheng Z-M, Cheng K-K, Hu Q-L, Liu H-J, Guo N-N, Liu D-H (2008). Effect of culture conditions on 3-hydroxypropionaldehyde detoxification in 1, 3-propanediol fermentation by *Klebsiella pneumoniae*. Biochem Eng J.

[55] Daniel R, Stuertz K, Gottschalk G (1995). Biochemical and molecular characterization of the oxidative branch of glycerol utilization by *Citrobacter freundii*. J Bacteriol.

[56] Luers F, Seyfried M, Daniel R, Gottschalk G (1997). Glycerol conversion to 1, 3-propanediol by *Clostridium pasteurianum*: cloning and expression of the gene encoding 1, 3-propanediol dehydrogenase. FEMS Microbiol Lett.

[57] Maru B, Bielen A, Constanti M, Medina F, Kengen S (2013). Glycerol fermentation to hydrogen by *Thermotoga maritima*: proposed pathway and bioenergetic considerations. Int J Hydrogen Energy.

[58] Tobimatsu T, Azuma M, Matsubara H, Takatori H, Niida T, Nishimoto K, Satoh H, Hayashi R, Toraya T (1996). Cloning, sequencing, and high level expression of the genes encoding adenosylcobalamin-dependent glycerol dehydrase of *Klebsiella pneumoniae*. J Biol Chem.

[59] Seyfried M, Daniel R, Gottschalk G (1996). Cloning, sequencing, and overexpression of the genes encoding coenzyme B_12_-dependent glycerol dehydratase of *Citrobacter freundii*. J Bacteriol.

[60] Claisse O, Lonvaud-Funel A (2001). Primers and a specific DNA probe for detecting lactic acid bacteria producing 3-hydroxypropionaldehyde from glycerol in spoiled ciders. J Food Prot.

[61] Wang W, Sun J, Hartlep M, Deckwer WD, Zeng AP (2003). Combined use of proteomic analysis and enzyme activity assays for metabolic pathway analysis of glycerol fermentation by *Klebsiella pneumoniae*. Biotechnol Bioeng.

[62] Saint-Amans S, Girbal L, Andrade J, Ahrens K, Soucaille P (2001). Regulation of carbon and electron flow in *Clostridium butyricum* VPI 3266 grown on glucose-glycerol mixtures. J Bacteriol.

[63] González-Pajuelo M, Meynial-Salles I, Mendes F, Andrade JC, Vasconcelos I, Soucaille P (2005). Metabolic engineering of *Clostridium acetobutylicum* for the industrial production of 1, 3-propanediol from glycerol. Metab Eng.

[64] González-Pajuelo M, Meynial-Salles I, Mendes F, Soucaille P, Vasconcelos I (2006). Microbial conversion of glycerol to 1, 3-propanediol: physiological comparison of a natural producer, *Clostridium butyricum* VPI 3266, and an engineered strain, *Clostridium acetobutylicum* DG1 (pSPD5). Appl Env Microbiol.

[65] Abbad-Andaloussi S, Guedon E, Spiesser E, Petitdemange H (1996). Glycerol dehydratase activity: the limiting step for 1, 3-propanediol production by *Clostridium butyricum* DSM 5431. Lett Appl Microbiol.

[66] Yamanishi M, Yunoki M, Tobimatsu T, Sato H, Matsui J, Dokiya A, Iuchi Y, Oe K, Suto K, Shibata N (2002). The crystal structure of coenzyme B_12_-dependent glycerol dehydratase in complex with cobalamin and propane-1, 2-diol. Eur J Biochem.

[67] Shibata N, Masuda J, Tobimatsu T, Toraya T, Suto K, Morimoto Y, Yasuoka N (1999). A new mode of *B* < *sub* > *12* <*/sub* > binding and the direct participation of a potassium ion in enzyme catalysis: x-ray structure of diol dehydratase. Structure.

[68] Masuda J, Shibata N, Morimoto Y, Toraya T, Yasuoka N (2000). How a protein generates a catalytic radical from coenzyme *B* < *sub* > *12* <*/sub* > : x-ray structure of a diol-dehydratase–adeninylpentylcobalamin complex. Structure.

[69] Hartmanis MG, Stadtman TC (1986). Diol metabolism and diol dehydratase in Clostridium glycolicum. Arch Biochem Biophys.

[70] Jiang W, Wang S, Yang Z, Fang B. B_12_-independent glycerol dehydratase and its reactivase from *Clostridia butyricum*: Optimizing cloning by uniform design logic. Eng life sci 2015.

[71] Brown KL, Marques HM (2001). Molecular modeling of the mechanochemical triggering mechanism for catalysis of carbon–cobalt bond homolysis in coenzyme *B* < *sub* > *12* <*/sub*>. J Inorg Biochem.

[72] O’Brien JR, Raynaud C, Croux C, Girbal L, Soucaille P, Lanzilotta WN (2004). Insight into the mechanism of the B_12_-independent glycerol dehydratase from *Clostridium butyricum*: preliminary biochemical and structural characterization. Biochemistry.

[73] Yakusheva M, Malahov A, Poznanskaya A, Yakovlev V (1974). Determination of glycerol dehydratase activity by the coupled enzymic method. Anal Biochem.

[74] Smiley K, Sobolov M (1962). A cobamide-requiring glycerol dehydrase from an acrolein-forming Lactobacillus. Arch Biochem Biophys.

[75] Toraya T, Banerjee R. Diol dehydratase and glycerol dehydratase. Chemistry and Biochemistry of B12. 1999:783–809.

[76] Toraya T (2000). Radical catalysis of B12 enzymes. structure, mechanism, inactivation, and reactivation of diol and glycerol dehydratases. Cell Mol Life Sci.

[77] Liao D-I, Dotson G, Turner I, Reiss L, Emptage M (2003). Crystal structure of substrate free form of glycerol dehydratase. J Inorg Biochem.

[78] Toraya T, Fukui S. Diol dehydrase. John Wiley and Sons 1982;12:233–262.

[79] Toraya T (1994). Diol Dehydrase and Glycerol Dehydrase, Coenzyme B ~ 1 ~ 2-Dependent Isozymes. Metal Ions Biol Sys.

[80] Banerjee R (1999). Chemistry and biochemistry of B_12_.

[81] Toraya T (1998). Recent structure-function studies of B_12_ coenzymes in diol dehydrase. Vitamin B.

[82] Barbirato F, Soucaille P, Bories A (1996). Physiologic Mechanisms Involved in Accumulation of 3-Hydroxypropionaldehyde during Fermentation of Glycerol by Enterobacter agglomerans. Appl Environ Microbiol.

[83] Kajiura H, Mori K, Tobimatsu T, Toraya T (2001). Characterization and mechanism of action of a reactivating factor for adenosylcobalamin-dependent glycerol dehydratase. J Biol Chem.

[84] Qi X, Sun L, Luo Z, Wu J, Meng X, Tang Y, Wei Y, Huang R (2006). Rational design of glycerol dehydratase: swapping the genes encoding the subunits of glycerol dehydratase to improve enzymatic properties. Chinese Sci Bull.

[85] Qi X, Guo Q, Wei Y, Xu H, Huang R (2012). Enhancement of pH stability and activity of glycerol dehydratase from *Klebsiella pneumoniae* by rational design. Biotechnol Lett.

[86] Gilis D, Rooman M (2000). PoPMuSiC, an algorithm for predicting protein mutant stability changes. Application to prion proteins. Protein Eng.

[87] Whittle E, Shanklin J (2001). Engineering Δ9-16: 0-Acyl Carrier Protein (ACP) Desaturase Specificity Based on Combinatorial Saturation Mutagenesis and Logical Redesign of the Castor Δ9-18: 0-ACP Desaturase. J Biol Chem.

[88] van den Heuvel RH, van den Berg WA, Rovida S, van Berkel WJ (2004). Laboratory-evolved vanillyl-alcohol oxidase produces natural vanillin. J Biol Chem.

[89] Siehl DL, Castle LA, Gorton R, Keenan RJ (2007). The molecular basis of glyphosate resistance by an optimized microbial acetyltransferase. J Biol Chem.

[90] Qi X, Chen Y, Jiang K, Zuo W, Luo Z, Wei Y, Du L, Wei H, Huang R, Du Q (2009). Saturation-mutagenesis in two positions distant from active site of a *Klebsiella pneumoniae* glycerol dehydratase identifies some highly active mutants. J Biotechnol.

[91] Bo L, Xiaolin X, Genlin Z, Qian L, Chun LK (2009). *pneumoniae* XJPD - Li glycerin dehydration enzyme gene point mutation and its performance study. J Shihezi University: Nat Sci Edn.

[92] Wang F, Qu H, Tian P, Tan T (2007). Heterologous expression and characterization of recombinant glycerol dehydratase from *Klebsiella pneumoniae* in *Escherichia coli*. Biotechnol J.

[93] Toraya T, Shirakashi T, Kosuga T, Fukui S (1976). Substrate specificity of coenzyme *B* < *sub* > *12* <*/sub* > -dependent diol dehydrase: glycerol as both a good substrate and a potent inactivator. Biochem Biophys Res Commun.

[94] Liao D-I, Reiss L, Turner I, Dotson G (2003). Structure of glycerol dehydratase reactivase: a new type of molecular chaperone. Structure.

[95] Toraya T, Fukui S (1977). Immunochemical Evidence for the Difference between Coenzyme-B_12_-Dependent Diol Dehydratase and Glycerol Dehydratase. Eur J Biochem.

[96] Tobimatsu T, Kajiura H, Toraya T (2000). Specificities of reactivating factors for adenosylcobalamin-dependent diol dehydratase and glycerol dehydratase. Arch Microbiol.

[97] Honda S, Toraya T, Fukui S (1980). In situ reactivation of glycerol-inactivated coenzyme B_12_-dependent enzymes, glycerol dehydratase and diol dehydratase. J Bacteriol.

[98] Mori K, Toraya T (1999). Mechanism of reactivation of coenzyme B_12_-dependent diol dehydratase by a molecular chaperone-like reactivating factor. Biochemistry.

[99] Bukau B, Horwich AL (1998). The Hsp70 and Hsp60 chaperone machines. Cell.

[100] Mori K, Tobimatsu T, Hara T, Toraya T (1997). Characterization, sequencing, and expression of the genes encoding a reactivating factor for glycerol-inactivated adenosylcobalamin-dependent diol dehydratase. J Biol Chem.

[101] Tobimatsu T, Kajiura H, Yunoki M, Azuma M, Toraya T (1999). Identification and expression of the genes encoding a reactivating factor for adenosylcobalamin-dependent glycerol dehydratase. J Bacteriol.

[102] Tobimatsu T, Hara T, Sakaguchi M, Kishimoto Y, Wada Y, Isoda M, Sakai T, Toraya T (1995). Molecular cloning, sequencing, and expression of the genes encoding adenosylcobalamin-dependent diol dehydrase of *Klebsiella oxytoca*. J Biol Chem.

[103] Toraya T (2002). Enzymatic Radical Catalysis: coenzyme B_12_-Dependent Diol Dehydratase. Chem Rec.

[104] Wanger O, Lee H, Frey P, Abeles D (1966). Studies on the mechanism on the action of cobamide coenzyme. J Biol Chem.

[105] Tobimatsu T, Azuma M, Hayashi S (1998). Nishimoto K-i, ToRAYA T. Molecular cloning, sequencing and characterization of the genes for adenosylcobalamin-dependent diol dehydratase of *Klebsiella pneumoniae*. Biosci Biotechnol Biochem.

[106] Toraya T (2003). Radical catalysis in coenzyme B_12_-dependent isomerization (eliminating) reactions. Chem Rev.

[107] Xu X, Zhang G, Wang LW, Ma BB, Li C (2009). Quantitative analysis on inactivation and reactivation of recombinant glycerol dehydratase from *Klebsiella pneumoniae* XJPD-Li. J Mol Catal B Enzym.

[108] Poppe L, Hull WE, Nitsche R, Graf T, Stupperich E, Rétey J (1999). (Hydroxyalkyl) cob (III) alamins as Competitive Inhibitors in Coenzyme B_12_-Dependent Enzymic Reactions: ^1^H-NMR Structure Analysis and Kinetic Studies with Glycerol Dehydratase and Diol Dehydratase. Helv Chim Acta.

[109] Berríos-Rivera SJ, Bennett GN, San KY (2002). Metabolic Engineering of *Escherichia coli*: increase of NADH Availability by Overexpressing an NAD < sup >+</sup > −Dependent Formate Dehydrogenase. Metab Eng.

[110] Nakamura CE, Whited GM (2003). Metabolic engineering for the microbial production of 1, 3-propanediol. Curr Opin Biotechnol.

[111] Zeng A-P, Biebl H. Bulk chemicals from biotechnology: the case of 1, 3-propanediol production and the new trends. In: Tools and Applications of Biochemical Engineering Science. Springer; 2002: 239–59. 10.1007/3-540-45736-4_11 11991182

[112] Laffend LA, Nagarajan V, Nakamura CE (1997). Bioconversion of a fermentable carbon source to 1, 3-propanediol by a single microorganism.

[113] Chase MW, Diaz-Torres M, Dunn-Coleman NS, Trimbur D (2000). Method for the recombinant production of 1, 3-propanediol.

[114] Emptage M, Haynie SL, Laffend LA, Pucci JP, Whited G (2009). Process for the biological production of 1, 3-propanediol with high titer.

[115] Tang X, Tan Y, Zhu H, Zhao K, Shen W (2009). Microbial conversion of glycerol to 1, 3-propanediol by an engineered strain of *Escherichia coli*. Appl Environ Microbiol.

[116] Skraly FA, Lytle BL, Cameron DC (1998). Construction and characterization of a 1, 3-propanediol operon. Appl Env Microbiol.

[117] Zhang X, Zhuge J. Construction of novel recombinant strain harboring glycerol dehydratase reactivating factor capable of producing 1, 3-propanediol. Bioeng Sci Rep. 2007;23(5). 18051862

[118] Bin Z, Yong W, Huiying F, Zhonggui M, Jian Z (2008). Genetic engineering reconstruction of *Klebsiella pneumoniae* producing 1,3-propanediol by the gene yqhD encoding 1,3-propanediol oxidoreductase isoenzyme. China Biotechnol.

[119] Huijin S, Fenghuan W, Pingfang T, Tianwei T. Two strategies implementation propylene glycol total expression of key enzyme genes. Journal of Beijing University of Chemical Industry 2007, 34(4).

[120] Bulthuis BA, Whited GM, Trimbur DE, Gatenby AA (2002). Method for the production of 1, 3-propanediol by recombinant organisms comprising genes for vitamin B_12_ transport.

[121] Jiang W, Li W, Hong Y, Wang S, Fang B. Cloning, Expression, Mutagenesis Library Construction of Glycerol Dehydratase, and Binding Mode Simulation of Its Reactivase with Ligands. *Appl Biochem Biotechnol* 2015:1-14. 10.1007/s12010-015-1906-6 26547853

[122] Biebl H (1991). Glycerol fermentation of 1, 3-propanediol by Clostridium butyricum. Measurement of product inhibition by use of a pH-auxostat. Appl Microbiol Biotech.

[123] Biebl H, Marten S (1995). Fermentation of glycerol to 1, 3-propanediol: use of cosubstrates. Appl Microbiol Biotechnol.

[124] Biebl H, Marten S, Hippe H, Deckwer W-D (1992). Glycerol conversion to 1, 3-propanediol by newly isolated clostridia. Appl Microbiol Biotech.

[125] Forsberg CW (1987). Production of 1, 3-propanediol from glycerol by *Clostridium acetobutylicum* and other *Clostridium* species. Appl Env Microbiol.

[126] Homann T, Tag C, Biebl H, Deckwer W-D, Schink B (1990). Fermentation of glycerol to 1, 3-propanediol by *Klebsiella* and *Citrobacter* strains. Appl Microbiol Biotech.

[127] Nakas J, Schaedle M, Parkinson C, Coonley C, Tanenbaum S (1983). System development for linked-fermentation production of solvents from algal biomass. Appl Environ Microbiol.

[128] Emptage M, Haynie SL, Laffend LA, Pucci JP, Whited GM (2006). Process for the biological production of 1, 3-propanediol with high titer.

[129] Nevoigt E, Stahl U (1997). Osmoregulation and glycerol metabolism in the yeast *Saccharomyces cerevisiae*. FEMS Microbiol Rev.

[130] Barbirato F, Larguier A, Conte T, Astruc S, Bories A (1997). Sensitivity to pH, product inhibition, and inhibition by NAD^+^ of 1, 3-propanediol dehydrogenase purified from *Enterobacter agglomerans* CNCM 1210. Arch Microbiol.

[131] Malaoui H, Marczak R (2000). Purification and characterization of the 1-3-propanediol dehydrogenase of *Clostridium butyricum* E5. Enzyme Microb Technol.

[132] Johnson E, Lin E (1987). *Klebsiella pneumoniae* 1, 3-propanediol: NAD^+^ oxidoreductase. J Bacteriol.

[133] Talarico TL, Dobrogosz WJ (1990). Purification and characterization of glycerol dehydratase from *Lactobacillus reuteri*. Appl Environ Microbiol.

[134] Deng WY, Wang F, Wang L, Lu AG, Meng JZ, Qi XH. Characteristic and Molecular Research of Glycerol Dehydratase. Adv Mat Res Trans Tech Publ. 2013: 892–895.

[135] Yuanyuan Z, Yang C, Baishan F (2004). Cloning and sequence analysis of the dhaT gene of the 1, 3-propanediol regulon from *Klebsiella pneumoniae*. Biotechnol Lett.

[136] Cao Y, Zheng Y, Fang B (2004). Optimization of polymerase chain reaction-amplified conditions using the uniform design method. J Chem Technol Biotechnol.

[137] Hongwen C, Baishan F, Zongding H (2005). Optimization of process parameters for key enzymes accumulation of 1, 3-propanediol production from *Klebsiella pneumoniae*. Biochem Eng J.

[138] Marçal D, Rêgo AT, Carrondo MA, Enguita FJ (2009). 1, 3-Propanediol dehydrogenase from *Klebsiella pneumoniae*: decameric quaternary structure and possible subunit cooperativity. J Bacteriol.

[139] Sulzenbacher G, Alvarez K, vanden Heuvel RHH, Versluis C, Spinelli S, Campanacci V, Valencia C, Cambillau C, Eklund H, Tegoni M (2004). Crystal Structure of E.coli Alcohol Dehydrogenase YqhD: evidence of a Covalently Modified NADP Coenzyme. J Mol Biol.

[140] Ma N. Molecular dynamics simulations of 1,3-propanediol oxidoreductase. 2011.

[141] Cao Y, Xia Q, Fang B (2006). Optimization of expression of dhaT gene encoding 1, 3-propanediol oxidoreductase from Klebsiella pneumoniae in *Escherichia coli* using the methods of uniform design and regression analysis. J Chem Technol Biotechnol.

[142] Juxiang Luo XW, Baishan Fang, QirongXia. Expression and Purification of Recombinant 1,3-propanediol oxidoreductase gene and part of the enzymatic properties. J Huaqiao University. 2008: 37.

[143] Seo J-W, Seo M-Y, Oh B-R, Heo S-Y, Baek J-O, Rairakhwada D, Luo LH, Hong W-K, Kim CH (2010). Identification and utilization of a 1, 3-propanediol oxidoreductase isoenzyme for production of 1, 3-propanediol from glycerol in *Klebsiella pneumoniae*. Appl Microbiol Biotechnol.

[144] Horng Y-T, Chang K-C, Chou T-C, Yu C-J, Chien C-C, Wei Y-H, Soo P-C (2010). Inactivation of dhaD and dhaK abolishes by-product accumulation during 1,3-propanediol production in *Klebsiella pneumoniae*. J Ind Microbiol Biotechnol.

[145] Zhuge B, Zhang C, Fang H, Zhuge J, Permaul K (2010). Expression of 1, 3-propanediol oxidoreductase and its isoenzyme in *Klebsiella pneumoniae* for bioconversion of glycerol into 1, 3-propanediol. Appl Microbiol Biotechnol.

[146] Zhao L, Ma X, Zheng Y, Zhang J, Wei G, Wei D (2009). Over-expression of glycerol dehydrogenase and 1, 3-propanediol oxidoreductase in *Klebsiella pneumoniae* and their effects on conversion of glycerol into 1, 3-propanediol in resting cell system. J Chem Technol Biotechnol.

[147] Ma Z, Rao Z, Xu L, Liao X, Fang H, Zhuge B, Zhuge J (2010). Expression of dha operon required for 1,3-PD formation in *Escherichia coli* and *Saccharomyces cerevisiae*. Current Microbiol.

[148] Devaux-Basseguy R, Bergel A, Comtat M. Potential applications of NAD(P)-dependent oxidoreductases in synthesis: A survey. In: Great Britain, BUTTERWORTH-HEINEMANN. 1997: 248–58.

[149] Wethmar M, Deckwer WD (1999). Semisynthetic culture medium for growth and dihydroxyacetone production by *Gluconobacter oxydans*. Biotechnol Tech.

[150] Zhang Hong-rui JP, Liu Shu-chen. Research Progress of Key Enzymes for Production of 1,3-Propanediol by Fermentation. Chem Bioeng. 2011: 28.

[151] Ruzheinikov SN, Burke J, Sedelnikova S, Baker PJ, Taylor R, Bullough PA, Muir NM, Gore MG, Rice DW (2001). Glycerol dehydrogenase: structure, specificity, and mechanism of a family III polyol dehydrogenase. Structure.

[152] Richter N, Breicha K, Hummel W, Niefind K (2010). The Three-Dimensional Structure of AKR11B4, a Glycerol Dehydrogenase from *Gluconobacter oxydans,* Reveals a Tryptophan Residue as an Accelerator of Reaction Turnover. J Mol Biol.

[153] Krauss O, Gore MG (1996). Refolding and reassociation of glycerol dehydrogenase from *Bacillus stearothermophilus* in the absence and presence of GroEL. Eur J Biochem.

[154] Musille P, Ortlund E (2014). Structure of glycerol dehydrogenase from Serratia. Acta Crystallographica Section F: Structural Biology Commun.

[155] Langendijk PS, Schut F, Jansen GJ, Raangs GC, Kamphuis GR, Wilkinson M, Welling GW (1995). Quantitative fluorescence in situ hybridization of Bifidobacterium spp. with genus-specific 16S rRNA-targeted probes and its application in fecal samples. Appl Environ Microbiol.

[156] Wu X, Xia Z, Yang X, Xue C, Lu W (2012). Molecular simulation of pyrroloquinoline quinine-dependent glycerol dehydrogenase in *Gluconobacter oxydans*. Mol Simulation.

[157] Li Zijun FB, Yang Zhongli, Liu Jia. Directed Evolution of Glycerol Dehydrogenase by Error Prone PCR. J Huaqiao University (Natur al Science) 2010:1000–1013.

[158] Pandey A, Iyengar L (2002). Chemical modification of specific active site amino acid residues of *Enterobacter aerogenes* glycerol dehydrogenase. J Enzyme Inhib Med Chem.

[159] Spencer P, Paine LJ, Scawen MD, Atkinson T, Gore MG (1990). Identification of a reversible structural transition in the metal-depleted glycerol dehydrogenase from *Bacillus stearothermophilus*. FEBS Lett.

[160] Guo YX, Wang SZ, Wang ZS, Chen R, and Fang BS. Chemical modification of the glycerol dehydrogenase by divalent metal ions. J Xiamen Univ Nat Sci 2011, 50:883–89.

[161] Wang S, Wang J, Zhou X, Guo Y, Fang B (2013). The improvement of stability, activity, and substrate promiscuity of glycerol dehydrogenase substituted by divalent metal ions. Biotechnol Bioprocess Eng.

[162] Leichus BN, Blanchard JS (1994). Isotopic analysis of the reaction catalyzed by glycerol dehydrogenase. Biochemistry.

[163] WEI Miao LH, Yan LI, YAN Ming XU (2011). Effects on growth and glycerol metabolism in E. coli by coexpression protein GldA and DhaKLM. Chinese Journal of. Bioprocess Eng.

[164] Zeng H, Fang B, Wang P, Zhang T, Baishan F. Process simulation design and techno-economic analysis of production technology of glycerol dehydrogenase. 2013.

[165] De Vries RP, Flitter SJ, De Vondervoort V, Peter J, Chaveroche MK, Fontaine T, Fillinger S, Ruijter GJ, D’Enfert C, Visser J (2003). Glycerol dehydrogenase, encoded by gldB is essential for osmotolerance in *Aspergillus nidulans*. Mol Microbiol.

[166] Matsuzawa T, Ohashi T, Hosomi A, Tanaka N, Tohda H, Takegawa K (2010). The gld1 + gene encoding glycerol dehydrogenase is required for glycerol metabolism in *Schizosaccharomyces pombe*. Appl Microbiol Biotechnol.

[167] Tang L, Yu J, Fang B (2011). Expression of glycerol dehydrogenase gene in Escherichia coli by codon optimization. Acta Microbiologica Sinica.

[168] Zhao L, Zheng Y, Ma X, Wei D (2009). Effects of over-expression of glycerol dehydrogenase and 1, 3-propanediol oxidoreductase on bioconversion of glycerol into 1, 3-propandediol by *Klebsiella pneumoniae* under micro-aerobic conditions. Bioprocess Biosyst Eng.

[169] Li M-h, Wu J, Liu X, Wei D-Z, Chen H (2010). Enhanced production of dihydroxyacetone from glycerol by overexpression of glycerol dehydrogenase in an alcohol dehydrogenase-deficient mutant of *Gluconobacter oxydans*. Bioresour Technol.

[170] Prodromidis M, Stalikas C, Tzouwara-Karayanni S, Karayannis M (1996). Determination of glycerol in alcoholic beverages using packed bed reactors with immobilized glycerol dehydrogenase and an amperometric FIA system. Talanta.

[171] Lapenaite I, Ramanaviciene A, Ramanavicius A (2006). Current trends in enzymatic determination of glycerol. Crit Rev Anal Chem.

[172] Zheng M, Zhang S (2011). Immobilization of glycerol dehydrogenase on magnetic silica nanoparticles for conversion of glycerol to value-added 1, 3-dihydroxyacetone. Biocatalysis Biotransform.

[173] Rocha-Martin J, Acosta A, Berenguer J, Guisan JM, Lopez-Gallego F (2014). Selective oxidation of glycerol to 1, 3-dihydroxyacetone by covalently immobilized glycerol dehydrogenases with higher stability and lower product inhibition. Bioresour Technol.

[174] Hongwen Chen JN (2010). Guo Chen, and Baishan Fang. Kinetic mechanisms of glycerol dehydrogenase and 1,3-propanediol oxidoreductase from *Klebsiella pneumoniae*. Chinese J Biotechnol.

[175] Takeuchi M, Okura I, Hasumi F (1991). Regeneration of NADH and hydrogenation of dihydroxyacetone by hydrogen with the combination of hydrogenase and glycerol dehydrogenase. J Mol Catalysis.

[176] Ichinose H, Kamiya N, Goto M (2005). Enzymatic redox cofactor regeneration in organic media: functionalization and application of glycerol dehydrogenase and soluble transhydrogenase in reverse micelles. Biotechnol Prog.

